# Stapling Cysteine[2,4] Disulfide Bond of α-Conotoxin LsIA and Its Potential in Target Delivery

**DOI:** 10.3390/md22070314

**Published:** 2024-07-14

**Authors:** Xin Sun, Jiangnan Hu, Maomao Ren, Hong Chang, Dongting Zhangsun, Baojian Zhang, Shuai Dong

**Affiliations:** 1 Key Laboratory of Tropical Biological Resources of Ministry of Education, School of Pharmaceutical Sciences, Hainan University, Haikou 570228, China; sunxin7605@163.com (X.S.); jnjnjn0521@163.com (J.H.); syphurmm@163.com (M.R.); zhangsundt@163.com (D.Z.); 2 Hainan Academy of Inspection and Testing, Haikou 570228, China; 65523885@163.com

**Keywords:** α-conotoxin, disulfide bond stapling, peptide-drug conjugates, fluorescent probe, target delivery

## Abstract

α-Conotoxins, as selective nAChR antagonists, can be valuable tools for targeted drug delivery and fluorescent labeling, while conotoxin-drug or conotoxin-fluorescent conjugates through the disulfide bond are rarely reported. Herein, we demonstrate the [2,4] disulfide bond of α-conotoxin as a feasible new chemical modification site. In this study, analogs of the α-conotoxin LsIA cysteine[2,4] were synthesized by stapling with five linkers, and their inhibitory activities against human α7 and rat α3β2 nAChRs were maintained. To further apply this method in targeted delivery, the alkynylbenzyl bromide linker was synthesized and conjugated with Coumarin 120 (AMC) and Camptothecin (CPT) by copper-catalyzed click chemistry, and then stapled between cysteine[2,4] of the LsIA to construct a fluorescent probe and two peptide-drug conjugates. The maximum emission wavelength of the LsIA fluorescent probe was 402.2 nm, which was essentially unchanged compared with AMC. The cytotoxic activity of the LsIA peptide-drug conjugates on human A549 was maintained in vitro. The results demonstrate that the stapling of cysteine[2,4] with alkynylbenzyl bromide is a simple and feasible strategy for the exploitation and utilization of the α-conotoxin LsIA.

## 1. Introduction

Cone snails (genus *Conus*) are carnivores that live in tropical oceans and feed by paralyzing fish, worms, or other mollusks with venom secretions. Cone snail venom contains a wide variety of polypeptides, which are called conotoxins. The diversity of conotoxins is exceptionally high. There are approximately 700 species of *Conus* in the world [1], and each type of snail can produce more than 3000 conotoxins [2]. It is estimated that there should be more than 2 million conotoxins in the world’s tropical oceans. The conotoxins were produced in the venom gland, salivary gland and accessory salivary gland [3,4]. Conotoxins target multiple receptors specifically, making them powerful tools for pharmacological and neuroscience research [5]. Conotoxins are a considerable treasure trove of marine peptide drugs and pharmacological tools development. Among the various conotoxins, α-conotoxins were the earliest to be discovered and the most studied. They act on nicotinic acetylcholine receptors (nAChRs), and are valuable probes for the studying of various nAChR subtypes, including their fine structures, physiological functions, and pharmacological functions [6]. α-Conotoxins are new drug sources for nAChR-related diseases such as neuralgia, addiction, and malignant tumors [7,8,9,10].

For the better application of conotoxins, it is necessary to achieve the connection between conotoxins and fluorescent molecules or drug molecules. The α-conotoxin ImI was used to actualize the targeted delivery of paclitaxel micelles to α7 nAChR-overexpressing breast cancer MCF-7 [11]. Later, this *α*-conotoxin ImI micelle system was utilized for targeting A549, a cell model of the non-small-cell lung cancer cell line [12]. The α-Conotoxin LtIA was covalently linked to a fluorescent molecule. The constructed fluorescent peptide can serve as a novel molecular probe for the study of the α3β2 nAChR subtype in live systems [13]. All of these chemical modifications were based on the N-termini of the conotoxins. However, for some conotoxins, the N-termini are important pharmacologically active sites, and they cannot withstand any chemical modification. Therefore, a new covalent attachment site needs to be developed.

Most studies in the past generally believed that the modification of disulfide bonds in conotoxins was limited to disulfide bond replacement based on bioisosterism [14,15,16,17,18]. However, our previous study found that the cysteine[2,4] disulfide bond of TxIB was an insertable modification site [19]. But it is not clear whether the disulfide bond insertion method can be extended to other conotoxins, as the sophisticated three-dimensional structure determines their activities. 

The α-conotoxin LsIA (17 amino acids in length) is a neuropeptide discovered from *Conus limpusi*, which is a potent antagonist of the α7 nAChR (IC_50_ = 10.1 nM) and α3β2 nAChR (IC_50_ = 10.3 nM) [20]. In this report, we continuously inserted different linkers into the cysteine[2,4] disulfide bond of the *α*-conotoxin LsIA, and found that the nAChR inhibitory activities were almost maintained. Specifically, the sulfhydryl side chains of the LsIA cysteine[2,4] residues were first reacted with five electrophilic molecules efficiently. Then, a disulfide bond of cysteine[1,3] was formed by normal iodine oxidation. The cysteine[1,3] linker stapling derivative was also synthesized as a control. The nAChR inhibitory activities of all the synthetic peptides were evaluated using a two-electrode voltage patch clamp. The results showed that the activities of the [2,4]-modified analogs were essentially maintained, while the activity of the [1,3]-modified analog was lost. With these results in hand, a disulfide stapling modification method was developed, which could combine the conotoxin with fluorescent or pharmaceutical molecules covalently. More concretely, the benzyl bromide linker was extended to an alkynylbenzyl bromide linker, which can be connected to any fluorescent molecules or drugs with an azide group by click chemistry. The α-conotoxin LsIA-AMC fluorescent probe and the LsIA-CPT peptide-drug conjugates were constructed by the same synthetic method described previously. The excitation and emission wavelengths of the modified fluorescent peptide were essentially unchanged compared to AMC, and the peptide-drug conjugates maintained their cytotoxic activities against A549 in vitro. 

## 2. Results and Discussion

### 2.1. Synthesis of Native LsIA and Its Cysteine [2,4]/[1,3] Modification Products

**Scheme 1:**
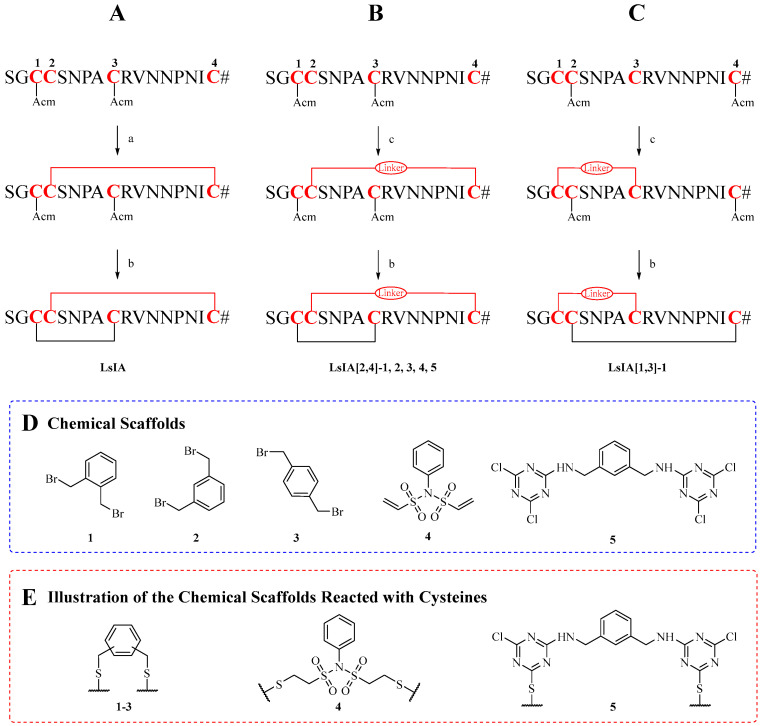
Synthetic routes of *α*-Conotoxin LsIA, LsIA[2,4]-1, 2, 3, 4, 5, and LsIA[1,3]-1 (# signifies C-term amidation). (**A**) Oxidative folding process of LsIA. (**B**) Cysteine[2,4] linker stapling of LsIA. (**C**) Cysteine[1,3] linker stapling of LsIA. (**D**) Structures of the linkers. *Reagents and conditions*: (a) K_3_[Fe(CN)_6_], Tris-HCl, MeCN, ultrapure water, rt, and 45 min; (b) I_2_, TFA, MeCN, ultrapure water, rt, 10 min, and vitamin C; (c) Chemical scaffolds **1**–**5**, NH_4_HCO_3_ buffer, MeCN, rt, and 65 min. (**E**) Illustration of the chemical scaffolds **1**, **2**, **3**, **4**, and **5** after reaction with the sulfhydryl groups of cysteines.

The resin-attached peptide was synthesized using standard Fmoc solid-phase synthesis technology, then cleaved from the resin and purified by preparative HPLC. The purity of each linear peptide was above 90%. As shown in Scheme 1A, the α-conotoxin LsIA was synthesized by a normal two-step oxidative folding method, which was conducted under a K_3_[Fe(CN)_6_] and I_2_ acidic solution [21]. The LsIA[2,4] series (Scheme 1B) and LsIA[1,3] series (Scheme 1C) were synthesized from the linear peptides with an acetamidomethyl (Acm) protecting group at cysteine[1,3] or cysteine[2,4] by first reacting with different linkers at the cysteine[2,4] or cysteine[1,3] positions, respectively. Linkers 1–5 were designed to verify the universality of the disulfide bond insertion method for the LsIA. For LsIA cysteine[2,4] modification products, the chemical scaffolds 1, 2, 3, 4, and 5 (Scheme 1D) were covalently reacted with the bare sulfhydryl groups of the unprotected cysteines under a weak alkaline condition in an NH_4_HCO_3_ buffer (Scheme 1E). Then the bicyclic final product was constructed by normal iodine oxidation under argon protection for 10 min. The LsIA[1,3]-1 was designed and synthesized by the same method as the LsIA[2,4] series to explore the interference of different modification sites on the nAChR inhibitory activity. All the products were purified by HPLC (>95% purity), identified by ESI-MS, and lyophilized for further use.

**Figure 1:**
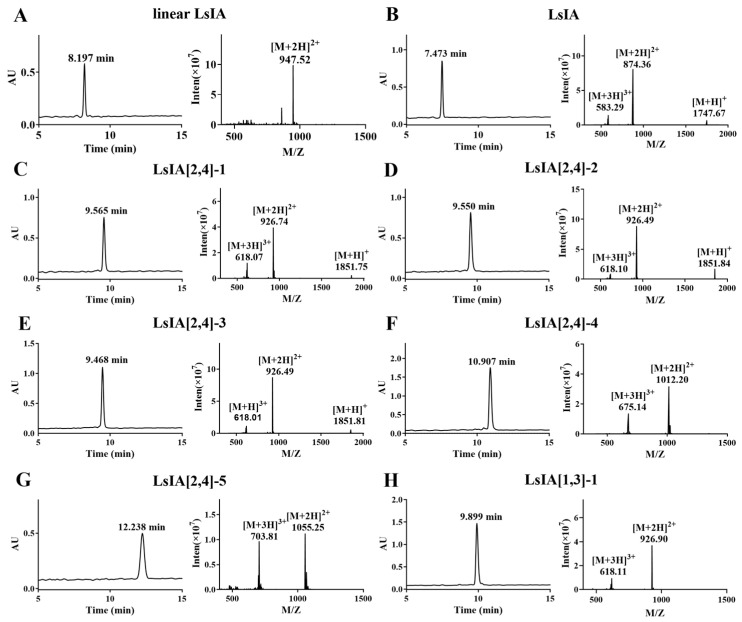
HPLC and ESI-MS profiles of linear LsIA (**A**), LsIA (**B**), and its analogs (**C**–**H**). The chromatographic conditions were 10% to 55% buffer B in buffer A with a flow rate of 1 mL/min at UV-214 nm over 20 min, and the column temperature was 40 °C. A = 0.1% trifluoroacetic acid in H_2_O and B = 0.1% trifluoroacetic acid in acetonitrile. AU, absorbance units.

The HPLC chromatograms and ESI-MS spectrograms of all the synthetic peptides are shown in Figure 1, and the theoretical and measured average molecular weights are shown in the Supplementary Materials—Table S1. The *α*-conotoxin LsIA was successfully synthesized with high purity. The retention time of the LsIA was 7.473 min. The mass-to-charge ratio (*m*/*z*) peaks of the LsIA were 1747.67 Da [M + H]^+^, 874.36 Da [M + 2H]^2+^, and 583.29 Da [M + 3H]^3+^, which is consistent with its theoretical average mass of 1746.97 Da (Figure 1B and Table S1). Compared to the molecular weight of the linear peptide, the LsIA decreased by 146.2 Da, which was caused by a two-stage mass deduction consistent with the formation of two disulfide bonds. Two hydrogen atoms were removed to form the first disulfide bond (−2.02 Da), and two Acm-protecting groups were removed to form the second disulfide bond (−144.18 Da). LsIA[2,4]-1, as shown in Figure 1C, was successfully synthesized by nucleophilic substitution and oxidation. The retention time was 9.565 min. ESI-MS was used to confirm that LsIA[2,4]-1 has an average molecular weight of 1851.13 Da with *m*/*z* peaks of 1851.75 [M + H]^+^, 926.74 Da [M + 2H]^2+^, and 618.07 Da [M + 3H]^3+^, which is consistent with its theoretical average mass of 1851.13 Da. Compared to the molecular weight of the linear peptide LsIA plus chemical scaffold **1**, LsIA[2,4]-1 decreased by 306 Da, which was caused by a two-stage mass deduction consistent with the insertion of linker **1** and the formation of a disulfide bond. Two hydrogen bromide molecules were removed to insert the chemical scaffold (−161.82 Da), and two Acm-protecting groups were removed to form the disulfide bond (−144.18 Da). The retention time of all the modified products increased by 2–5.5 min compared with the α-conotoxin LsIA, indicating an increase in their hydrophobicity (Figure 1).

### 2.2. The nAChRs Inhibitory Activity Evaluation of LsIA and its Analogs

**Figure 2:**
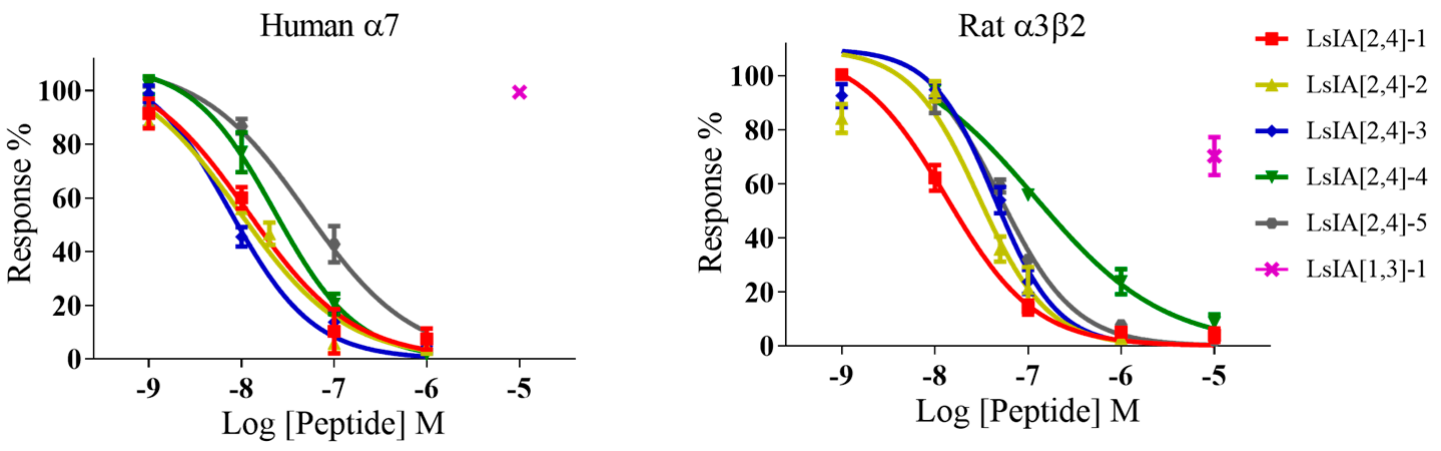
Concentration–response curves of LsIA analogs on human α7 and rat α3β2 nAChRs. Each data point was presented as the mean ± SEM of 3–10 separated oocytes.

Herein, the nAChR inhibitory activities of the LsIA[1,3] and [2,4] modification analogs were evaluated by a two-electrode voltage clamp method at room temperature. The concentration–response curves of these analogs on the α7 and α3β2 nAChRs are shown in Figure 2.

The IC_50_ values of the LsIA and its analogs on the nAChRs are listed in Table 1. The LsIA has IC_50_ values of 10.1 nM and 10.3 nM on the α7 and α3β2 nAChRs, respectively [20]. The inhibitory activities on the α7 and α3β2 nAChRs of all cysteine[2,4]-modified products were maintained, while the cysteine[1,3]-modified product LsIA[1,3]-1 did not show potent blocking activity even at a concentration of 10 μM, which was consistent with our previous study [19]. LsIA[2,4]-1 has the minimum IC_50_ value (11.9 nM for α7 nAChR and 13.3 nM for α3β2 nAChR) compared with the other four cysteine[2,4]-modified derivatives. The IC_50_ values for the *α*7 nAChR of LsIA[2,4]-1,2,3 slowly decreased along with the increased distance between the two sulfur atoms of cysteine[2,4], while for the α3β2 nAChR, the inhibitory activities of the three derivatives followed the opposite pattern. LsIA[2,4]-3,4 both showed a five-fold selectivity, preferring α7 nAChR to α3β2 nAChR, while the IC_50_ values of LsIA[2,4]-5 against the two nAChRs were almost the same. 

The disulfide bond is vital for the structure, stability, and pharmacological activity of α-conotoxins [22]. The insertion of small frameworks into disulfide bonds is widely studied in peptides. There are numerous chemical compounds that can react with the sulfhydryl group selectively [23]. Moreover, some perfluoroarene frameworks may improve their penetration ability [24]. This study on conotoxin disulfide bond modification is mainly about disulfide bond substitution, including thioether substitution, diselenide substitution, saturated carbon-carbon substitution, alkene substitution, amide substitution, and 1,2,3-triazole substitution, etc. [5]. Diselenide bond substitutions maintained or improved its blocking activity on the nAChRs [25], and 1,2,3-triazole replacement increased its stability [18]. At the same time, some studies revealed similar results to our studies, showing that a 1,3-position substitution decreased its blocking activity [26,27]. In this research, the LsIA[2,4] derivatives exhibited a strong potency on the nAChRs even when stapling with a relatively large molecule such as linkers **4** and **5** (Scheme 1D), which indicated that the cysteine[2,4] of LsIA is a modifiable site for a chemical frameworks insertion.

### 2.3. CD Spectroscopy Assays

**Figure 3:**
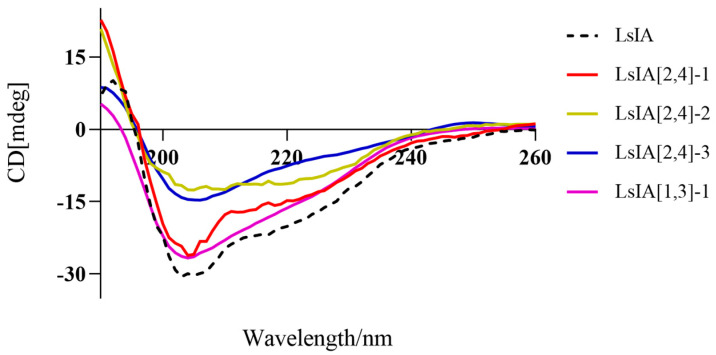
Circular dichroism (CD) spectra of LsIA and its analogs.

CD spectroscopy was used to study the conformational changes between the LsIA and its analogs LsIA[2,4]-1, LsIA[2,4]-2, LsIA[2,4]-3, and LsIA[1,3]-1. As shown in Figure 3, the positive Cotton effect around 190 nm and negative Cotton effect around 200 nm to 220 nm of the LsIA and its analogs indicated the presence of an α-helix and a β-sheet secondary structure. The CD spectra of LsIA[2,4]-1 and LsIA[2,4]-2 were similar to the LsIA, which showed a W-shaped pattern with negative ellipticities at 206 and 220 nm. The similarity of the CD spectra indicated the conserved three-dimensional structures, which are consistent with the maintenance of the nAChR blocking activities of LsIA[2,4]-1-2 (Table 1). Meanwhile, the CD spectra of LsIA[2,4]-3 showed weak negative ellipticities at 220 nm, and the activity and selectivity for α7 and α3β2 were changed. However, LsIA[1,3]-1 showed obviously different spectra from the LsIA with only one negative band at 206 nm, which reflected the different three-dimensional structures between them. The variation of the three-dimensional structure may be the reason for the loss of the nAChR blocking activity of LsIA[1,3]-1. The secondary structure contents of the LsIA and its analogs are shown in the supporting information (Supplementary Materials—Table S3).

### 2.4. Construction of Fluorescent Probe and Peptide-Drug Conjugates

Click chemistry has become a prime example of current bioorthogonal reactions with the advantages of high reaction efficiency, mild reaction conditions, remarkable chemo- and regioselectivities, and excellent functional group compatibility under mild reaction conditions [28,29,30]. Herein, a small benzyl bromide molecule with a terminal alkyne was synthesized as the linker. On the one hand, the alkynyl group can react efficiently with an azide through the click chemistry. On the other hand, the benzyl bromine can react with the thiol groups on the LsIA. In fact, all the chemical scaffolds **1**–**5** can be designed to link drugs or fluorescent molecules with the LsIA. Linker **6** was designed based on linker 2 because it is easily synthesized and structurally symmetrical. The alkynylbenzyl bromide linker **6** was synthesized under the conditions outlined in the supporting material (Supplementary Materials—Scheme S1) in gram scale. All synthetic steps were described in the experimental section, and the structures of the compounds were determined by ESI-MS, ^1^H NMR (proton nuclear magnetic resonance), and ^13^C NMR (carbon nuclear magnetic resonance). Their purity was confirmed by RP-HPLC (Reversed-phase high-performance liquid chromatography).

**Scheme 2:**
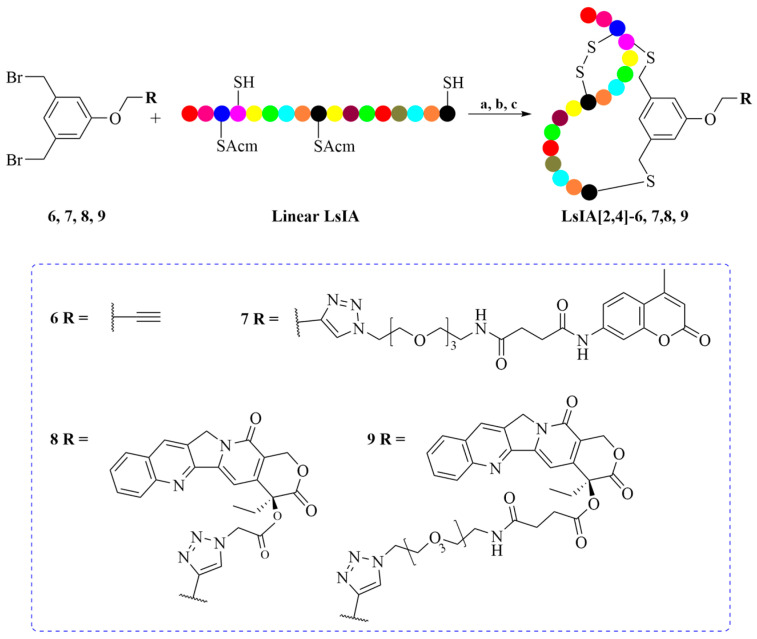
Synthetic route of LsIA[2,4]-6, 7, 8, and 9. *Reagents and conditions*: (a) CuSO_4_·5H_2_O, sodium ascorbate, MeCN-H_2_O, rt, and overnight; (b) NH_4_HCO_3_ buffer, MeCN, rt, and 65 min; (c) I_2,_ TFA, MeCN, H_2_O, rt, and 10 min.

**Figure 4:**
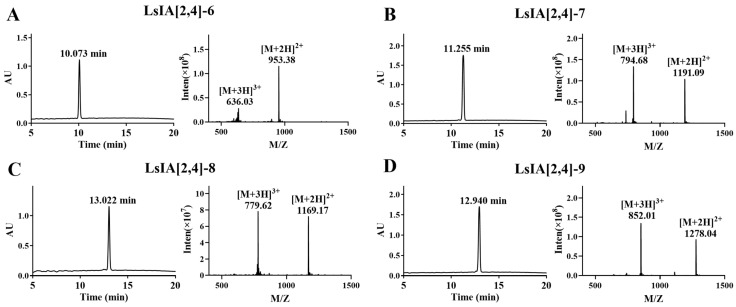
HPLC and ESI-MS profiles of LsIA[2,4]-6 (**A**), LsIA[2,4]-7 (**B**), LsIA[2,4]-8 (**C**), and LsIA[2,4]-9 (**D**). The chromatographic conditions were 10% to 55% buffer B in buffer A with a flow rate of 1 mL/min at UV-214 nm over 20 min, and the column temperature was 40 °C. A = 0.1% trifluoroacetic acid in H_2_O and B = 0.1% trifluoroacetic acid in acetonitrile. AU, absorbance units.

In this experiment, AMC and CPT were selected for the synthesis of the fluorescent probe and peptide-drug conjugate (PDC), respectively. Linker **6** can be easily connected to the side chain of cysteine[2,4] by nucleophilic substitution, and then the alkynyl group could be connected with any synthetic or commercial azides by click chemistry. In this project, considering the high cost of the peptides, we first connected the azido-derivatized AMC and CPT with the alkynyl linker **6** to make the precursor compounds **7**–**9** (Supplementary Materials—Scheme S2). The compounds **6**–**9** were then reacted with the sulfhydryl groups of cysteine[2,4], and followed by iodine oxidation to construct LsIA[2,4]-6, 7, 8, and 9 (Scheme 2). This reaction route will reduce the waste of the linear LsIA. Fluorescent modification of conotoxins mainly relied on the N-terminal nucleophilic substitution with an N-hydroxysuccinimide (NHS) functional group previously [13], which is susceptible to the amino groups of lysine. Using the sulfhydryl group as the linkage site proved to be an efficient method for conotoxin folding and fluorescent labeling with fewer by-products. The synthetic routes of compounds **6**–**9** are shown in the supporting information (Supplementary Materials—Schemes S1 and S2). The molecular weight and purity of the intermediate LsIA[2,4]-6, fluorescent probe LsIA[2,4]-7, and PDCs LsIA[2,4]-8-9 were determined by ESI-MS and HPLC, and the molecular weight was consistent with the theoretical molecular weight (Figure 4 and Supplementary Materials—Table S2).

### 2.5. Fluorescence Spectrum Evaluation of the LsIA Fluorescent Probe

**Figure 5:**
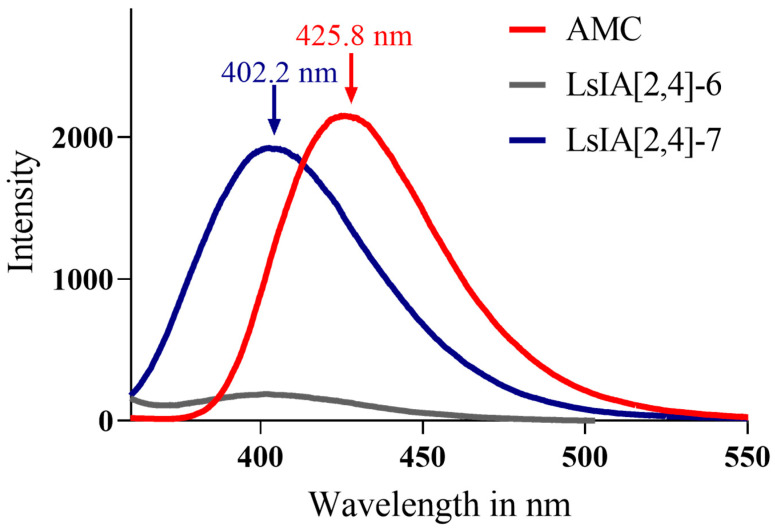
Fluorescence emission spectra of AMC (red), LsIA[2,4]-6 (grey), and LsIA[2,4]-7 (blue). Concentration: 100 μM, excitation at 340 nm.

The fluorescent molecule AMC, with an emission wavelength of 390–480 nm at the UV-excitable blue fluorescence, is suitable for fluorescence measurements of biological samples [31]. The fluorescence excitation and emission spectra of LsIA[2,4]-6, LsIA[2,4]-7, and AMC were evaluated (Figure 5). A noticeable change in fluorescence intensity was observed for LsIA[2,4]-7 compared with LsIA[2,4]-6. The LsIA[2,4]-7 conjugated with AMC showed almost the same fluorescence emission intensity as AMC. However, the LsIA[2,4]-6 without AMC showed no fluorescence emission. These results proved the successful connection between the LsIA and AMC without fluorescence quenching. But the maximum emission wavelength of AMC has a little blueshift from 425.8 nm to 402.2 nm after conjugating with the LsIA.

### 2.6. Evaluation of the Cytotoxic Activity of PDC In Vitro

**Figure 6:**
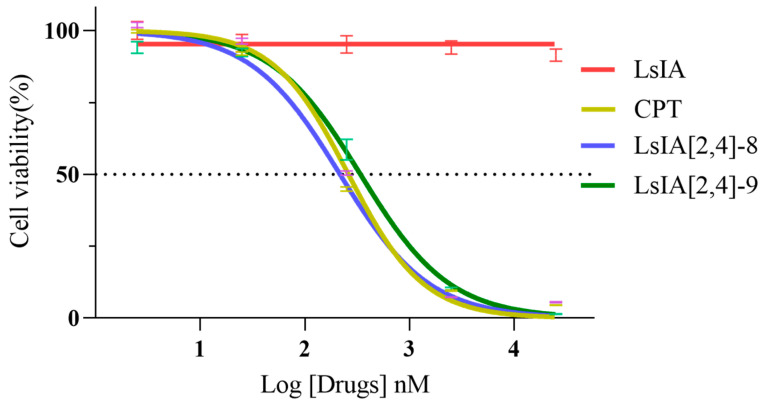
CCK8 experiment to evaluate the cytotoxicity of LsIA, CPT, LsIA[2,4]-8, and LsIA[2,4]-9 at 2.5 nM, 25 nM, 250 nM, 2.5 μM, and 25 μM. Each data point represents mean *±* SEM (*n* = 5).

In this project, a non-small-cell lung cancer cell line (A549) with high expression of the α7 nAChR was selected as the cell model [32]. The cell cytotoxicity of the LsIA, CPT, LsIA[2,4]-8, and LsIA[2,4]-9 for A549 was evaluated using a CCK8 assay with five drug concentrations between 2.5 nM and 25 μM (Figure 6). The IC_50_ values of LsIA[2,4]-8 and LsIA[2,4]-9 were 215.9 nM and 338.4 nM, respectively, which were almost the same as the CPT (259.9 nM). Furthermore, the LsIA did not inhibit tumor growth even at a concentration of 25 μM (Figure 6). This result indicated that the α-conotoxin modified CPT did not affect its antitumor ability. The α-conotoxin, as the nAChRs selective peptide [33], has an excellent potency for use in the application of targeted antitumor drug delivery in vivo. CPT is a topoisomerase inhibitor and showed potent antitumor activity [34]. However, poor water solubility and severe side effects have restricted its application in clinical practice [35]. Conjugating the water-soluble and nAChR-targeted LsIA with the CPT will improve the selectivity and water solubility of CPT.

## 3. Materials and Methods

### 3.1. Chemicals, Materials, and Instruments

The acetonitrile (MeCN, chromatographically pure grade) was purchased from Sigma-Aldrich (St. Louis, MO, USA). Trifluoroacetic acid (TFA, chromatographically pure grade) was purchased from Aladdin (Shanghai, China). All the other reagents used for the peptide chemical synthesis were obtained from GL Biochem (Shanghai, China) and Applied Biosystem (Foster City, CA, USA). Compounds **1**–**3** were purchased from Aladdin (Shanghai, China), and Compounds **4**–**9** were synthesized according to the procedures outlined in the supporting material (Supplementary Materials—Schemes S1 and S2). All the reagents, unless prompted, were analytical grade and obtained commercially. Mass spectra were obtained on an UPLC-ESI-MS (Acquity H Class-Xevo TOD, Waters, Milford, MA, USA). Preparative HPLC was performed using a Waters Prep 150 instrument with an autosampler (Waters 2707), a pump (Waters 2535, Quaternary Gradient Module), an UV/visible detector (Waters 2489), and a YMC-Pack ODS-AQ column (250 × 20 mm, 5 µm, YMC, Kyoto, Japan). The reversed-phase high-performance liquid chromatography (RP-HPLC) analysis was performed using a Waters e2695 system and an UV/visible detector (Waters 2489) with a Waters XBridge C18 column (250 × 4.6 mm, 5 μm, Waters, Milford, MA, USA). The RNA transcription and purification kits were purchased from Invitrogen (Invitrogen, Vilnius, Republic of Lithuania). All the reagents unless mentioned were of analytical grade and obtained commercially. *Xenopus laevis* (*X. laevis*) was purchased from Nasco (Fort Atkinson, WI, USA). The protocol to obtain *X. laevis* oocytes was approved by the Ethics Committee of Hainan University and strictly adhered to the guidelines for the care and use of laboratory animals for this study. Oocytes from *X. laevis* were surgically removed and cultured as previously described [36,37,38]. The DMEM-H (Dulbecco’s modified Eagle’s medium–high glucose) and penicillin/streptomycin solutions were purchased from Sangon Biotech (Shanghai, China). The FBS (fetal bovine serum) was purchased from Procell (Wuhan, China). The Cell Counting Kit-8 was purchased from Biosharp (Hefei, China).

### 3.2. Synthesis of LsIA 

The sequence of the linear conotoxin LsIA is SGCCSNPACRVNNPNIC# (# signifies C-term amidation). The linear peptides were synthesized on a MBHA resin through a standard Fmoc solid-phase synthesis and cleaved from the resin by treatment with the cleavage mixtures (TFA–water–phenol–thioanisole–EDT, 82.5:5:5:5:2.5, v:v:v:v:v) for 1.5–2 h. The first and third cysteine residues of the LsIA[2,4] were protected by Acm, and the second and fourth cysteine residues of the LsIA[1,3] were protected by Acm. The crude-precipitated peptide was washed with ether (−20 °C) and recycled by centrifuging at 15,000× *g* for 10 min at 4 °C three times.

The linear peptide LsIA[2,4] was then folded by the two-step oxidative folding reaction [21]. The linear peptide LsIA[2,4] (20 mg, 0.01 mmol) was added to a solution of K_3_[Fe(CN)_6_] (330 mg, 1.00 mmol) and Tris-HCl (606 mg, 3.86 mmol) in MeCN-H_2_O (9:1, 100 mL). The reaction mixture was stirred at room temperature in the open flask for 45 min to bridge the monocyclic product, then purified by preparative HPLC (5–45% buffer B with a flow rate of 8 mL/min within 40 min at UV-214 nm, where buffer A was 0.1% TFA in water and buffer B was 0.1% TFA in acetonitrile) and characterized by UPLC-MS. The purified monocyclic product solution (25 mL) was used directly for the next step to construct the final bicyclic product by iodine oxidation. The monocyclic product solution was added to the solution of I_2_ and TFA (20 mg I_2_ and 1 mL TFA in 70 mL 10% MeCN-H_2_O) under a nitrogen atmosphere. After stirring for 10 min, a saturated vitamin C solution was added dropwise to terminate the reaction, with the yellow solution turning colorless. The bicyclic product was separated by preparative HPLC (5–45% buffer B with a flow rate of 8 mL/min within 40 min at UV-214 nm, where buffer A was 0.1% TFA in water and buffer B was 0.1% TFA in acetonitrile) and characterized by UPLC-MS.

### 3.3. General Procedure for the Synthesis of Modified Peptide Products

An NH_4_HCO_3_ buffer (16 mL, 0.2 M, and pH 8.2) was added to the solution of the LsIA[2,4] (20 mg in 4 mL H_2_O, 1 equiva) under a nitrogen atmosphere. After stirring for 2 min, the MeCN solution of compounds **1**–**9** (4 equiva, 4 mM) was added dropwise. The reaction mixture was stirred at room temperature for 65 min, then trifluoroacetic acid (0.1% in water,160 mL) was poured in to terminate the reaction. The product was separated by preparative HPLC (5–45% buffer B with a flow rate of 8 mL/min at UV-214 nm within 40 min, where buffer A was 0.1% TFA in water and buffer B was 0.1% TFA in acetonitrile) and characterized by UPLC-MS. The next reaction step was consistent with the iodine oxidation for the *α*-conotoxin LsIA. 

### 3.4. Circular Dichroism Spectroscopy 

A Jasco J-810 spectropolarimeter was employed to test the CD spectra of the native LsIA and its analogs. A cell with a capacity of 500 μL and a path length of 0.1 cm was used. The concentration of peptides was 0.15 mg/mL in water. The spectra were measured in the far UV region (190–240 nm) using an average of 10 scans. The experimental parameters of the scanning speed were 100 nm/min, a 0.5 s response time, 100 millidegrees sensitivity range, and a 1 nm step resolution to maintain stable measurement conditions, while the flow of nitrogen was maintained at 15 mL/min. All experiments were carried out at room temperature (26 °C). The data were analyzed and processed by DichroWeb [39].

### 3.5. Fluorescence Spectra

A Hitachi F-4700 fluorescence spectrophotometer was employed to record the AMC, LsIA[2,4]-6, and LsIA[2,4]-7 fluorescence spectra. Fluorescence emission spectra were screened between 300 and 800 nm at an excitation wavelength of 340 nm. All samples were tested at a concentration of 100 μM.

### 3.6. nAChRs Expressed on Xenopus Oocytes

The plasmids of the rat (α3, β2) and human (α7) nAChR subunits were linearized by the corresponding enzymes for in vitro cRNA transcription using the mMessage mMachine kit (Ambion, Austin, TX, USA). The cRNA was purified by the MEGA Clear Kit (Ambion). The cRNA of the nAChR subunits was injected into the *Xenopus* oocytes (within 59.8 nL). Although the physiological and pharmacological properties of the α3β4 nAChR and α4β2 nAChR isoforms can be affected by the ratio of α to β subunits [40,41], a study in our group found that the stoichiometry of the α3 and β2 subunits has no effect on the α3β2 nAChR. Therefore, for the α3β2 nAChR, the cRNA was mixed in a ratio of 1:1. The injected oocytes were then incubated at 17 °C in an ND-96 buffer with the mixed antibiotic (10 mg/L penicillin, 10 mg/L streptomycin, and 100 mg/L gentamicin) for 1–5 days. Finally, the oocytes expressed rα3β2 and hα7 nAchRs which were harvested. 

### 3.7. Electrophysiological Activity on Different nAChRs

The samples (>95% purity) were dissolved in an ND-96 solution (pH 7.5), respectively (ready for use). Then the oocyte was fixed in the oocyte chamber (a cylindrical well, 50 µL in volume), and ND-96 solution of 1 µM atropine and 0.1 mg/mL bovine serum albumin was gravity-perfused at a rate of 2 mL/min. The membrane currents of the *Xenopus* oocytes expressing different subtypes of nAChRs were recorded by TEVC. The membrane potential of the oocytes was clamped at −70 mV. Two seconds of 100 µM ACh and 58 s of ND-96 were applied to the oocyte each time, which was repeated until a stable baseline was obtained three times continuously. Next, the oocytes were incubated with either ND-96 or different concentrations of conotoxins in ND-96 for 5 min, followed by ACh stimulation (2 s ACh and 58 s ND-96) repeatedly. The membrane currents were recorded by TEVC at 26 °C. All the nAChR inhibitory activities were tested by this method. The concentration–response curves for all peptides were obtained by nonlinear regression analysis: % response = 100/(1 + ([peptide]/IC_50_) ^nH^), where nH is the hill slope, and IC_50_ indicates the inhibitory concentration of the antagonist required to produce 50% inhibition of the agonist response. All data represent the mean ± S.E.M. of at least three independent experiments, and were statistically analyzed using Prism 8.0 software (GraphPad Software, San Diego, CA, USA).

### 3.8. Cell Line and Cytotoxicity Assay

The human non-small-cell lung cancer cell line A549 was purchased from the Conservation Genetics Chinese Academy of Sciences Kunming Cell Bank. A549 cells were cultured with phenol red DMEM-H supplemented with 10% (v/v) FBS, 100 unit/mL penicillin, and 100 µg/mL streptomycin at 37 °C in a 5% CO_2_ cell incubator. A CCK-8 viability assay was used in this study to assess the antitumor activity of the CPT and PDCs (LsIA[2,4]-8 and LsIA[2,4]-9). A549 cells were cultured and inoculated into 96-well plates at a density of 3 × 10^3^ cells per well. After 24 h of culture at 37 °C in a 5% CO_2_ cell incubator, the CPT and PDCs were added at five concentrations (2.5 nM, 25 nM, 250 nM, 2.5 μM, and 25 μM) and incubated for 72 h. Five replicates were set for each concentration. After discarding the original medium, 100 µL of a complete DMEM medium containing 10% CCK-8 reagent was added to each well and incubated at 37 °C for 1–2 h. Then, the absorbance (Abs) value was detected at a wavelength of 450 nm using a SpectraMax M2 microplate reader (Molecular Devices, Sunnyvale, CA, USA) to calculate the effect of the drug on cell viability. The cell viability was calculated as the following: inhibition rate (%) = ((Abs control group − Abs experiment group)/(Abs control group − Abs blank group)) × 100%. Cell survival was quantified as follows: survival (%) = ((Abs experimental group − Abs blank group)/(Abs control group − Abs blank group)) × 100% using GraphPad Prism 8 software (San Diego, CA, USA).

## 4. Conclusions

Peptide-drug conjugation, as an efficient strategy for targeted drug delivery, can improve drug utilization efficiency, especially for chemotherapy drugs. α-Conotoxins, as selective antagonists of nAChRs, can be valuable tools for targeted drug delivery toward cancers, such as non-small-cell lung cancer cells (NSCLC) and glioblastoma cells where nAChRs are over-expressed. In contrast, there are a few studies about conotoxin-drug conjugates.

In summary, a practical linker based on click chemistry with an alkynyl group was rationally constructed and applied to the fluorescent molecule and drug connection of the α-conotoxin LsIA. Firstly, six cysteine[2,4] or [1,3] disulfide bond inserted modification analogs were synthesized. The cysteine[2,4]-modified analogs showed nanomolar inhibitory activities on the α7 and α3β2 nAChR subtypes which were comparable to the α-conotoxin LsIA, while the cysteine[1,3]-modified analog lost its nAChR inhibitory activity. These results demonstrated that the cysteine[2,4] disulfide bond is a modifiable site for the LsIA. Then the alkynylbenzyl bromide linker was designed, synthesized, and connected to a fluorescent molecule (AMC) and anticancer drug (CPT) by an azido-alkyne click reaction. With the AMC-benzyl bromide and CPT-benzyl bromide in hand, the α-conotoxin LsIA fluorescent probe and peptide conjugated drugs were prepared, respectively. As shown in Figure 2 and Table 1, even with the α-conotoxin LsIA cysteine[2,4] stapling with the big chemical scaffolds **4** and **5**, their inhibitory activities against the α7 and α3β2 nAChRs were still at nanomolar scale. In addition, the long linkers between the peptide and drugs normally showed no significant effects on activities in many reports [42,43]. Based on the above two points, the activities of the conjugates should be maintained, and the activities of the conjugates were briefly evaluated, and showed good inhibitory activities against the two nAChRs. The emission wavelengths of the modified fluorescent peptide were essentially unchanged compared with AMC. In vitro cytotoxic activity results against A549 showed that the constructed PDCs maintained their activities compared with CPT, with IC_50_ values ranging from 215.9 nM to 338.4 nM. Overall, the above work illustrates the feasibility of this transformation strategy in targeted drug delivery and fluorescent labeling. Next, we will further explore the specific application method of this strategy in vivo.

## Figures and Tables

**Table 1:** Half-maximal inhibitory concentration (IC_50_) values from concentration–response curves for LsIA and its analogs at human α7 and rat α3β2 nAChR subtypes *^a^*.

Reference	α3β2 (IC_50_)	α7 (IC_50_)	LsIA Analogs
[20]	10.3 nM (8.8–12.1 nM)	10.1 nM (8.7–11.6 nM)	LsIA
This work	13.3 nM (10.6–16.7 nM)	11.6 nM (8.1–16.7 nM)	LsIA[2,4]-1
This work	29.7 nM (19.4–45.4 nM)	9.4 nM (7.3–12.2 nM)	LsIA[2,4]-2
This work	43.6 nM (34.1–55.9 nM)	7.6 nM (5.9–9.7 nM)	LsIA[2,4]-3
This work	119.7 nM (89.5–160.2 nM)	22.8 nM (16.4–31.6 nM)	LsIA[2,4]-4
This work	50.0 nM (43.3–57.6 nM)	49.8 nM (30.9–80.3 nM)	LsIA[2,4]-5
This work	>10 μM	>10 μM	LsIA[1,3]-1

*^a^* Numbers in parentheses are 95% confidence intervals. Mean of data from *n* = 3–10 experiments.

## Data Availability

Data will be made available upon request.

## Supplementary Materials

The following are available online at https://www.mdpi.com/article/10.3390/md22070314/s1, Mass and secondary structure data of the LsIA and its derivatives, Table S1: Theoretical and average calculated masses from observed masses (Da) of LsIA and its derivatives, Table S2: Theoretical and average calculated masses from observed masses (Da) of fluorescent probe and PDCs, Table S3: Secondary structure contents of LsIA and its analogs; the synthesis procedures and characterization of Linkers 4~6, AMC derivative, and CPT derivatives.
