# Supplementary material for: Stapling Cysteine[2,4] Disulfide Bond of α-Conotoxin LsIA and Its Potential in Target Delivery
Source: Mar Drugs. 2024 Jul 14;22(7):314. doi: 10.3390/md22070314 (PMC11278161; doi:10.3390/md22070314)
Supplement: Supplementary file 1 [file marinedrugs-22-00314-s001.zip › marinedrugs-3084103-supplementary.pdf]

Supporting Information

Stapling Cysteine [2,4] Disulfide Bond of α-Conotoxin LsIA and its Preliminary Application in Target Delivery

Xin Sun[1, †], Jiangnan Hu[1, †], Maomao Ren[1], Hong Chang[2], Dongting Zhangsun[1], Baojian Zhang[1, *], and Shuai Dong[1, *]

[1] Key Laboratory of Tropical Biological Resources of Ministry of Education, School of Pharmaceutical Sciences, Hainan University, Haikou 570228, China; sunxin7605@163.com (X.S.); JNJNJN0521@163.com (J.H.); syphurmm@163.com (M.R.); zhangsundt@163.com (D.Z.)

[2] Hainan Academy of Inspection and Testing, Haikou 570228, China; 375552783@qq.com (H.C.)

* Correspondence: zhangbaojian96@163.com (B.Z.); dongshuai_1024@163.com (S.D.)

Table of contents

1. Mass and secondary structure data of LsIA and its derivatives....................2
2. Synthesis of Linker 4~6, AMC derivative and CPT derivatives....................2
    2.1. General procedures and apparatus....................2
    2.2. Synthesis and characterization....................3
    2.3. HPLC, Mass and NMR analyses....................7
3. References....................17

1. Mass and secondary structure data of LsIA and its derivatives

Table S1. Theoretical and average calculated masses from observed masses (Da) of LsIA and its derivatives.

Peptides	Theoretical (Da)	Average calculated (Da)
Linear LsIA	1893.16	1893.02
LsIA	1746.97	1746.73
LsIA[2,4]-1	1851.13	1851.13
LsIA[2,4]-2	1851.13	1851.02
LsIA[2,4]-3	1851.13	1850.92
LsIA[2,4]-4	2022.31	2022.39
LsIA[2,4]-5	2108.16	2108.44
LsIA[1,3]-1	1851.13	1851.54

Table S2. Theoretical and average calculated masses from observed masses (Da) of fluorescent probe and PDCs.

Peptides	Theoretical (Da)	Average calculated (Da)
LsIA[2,4]-6	1905.17	1904.97
LsIA[2,4]-7	2380.68	2380.42
LsIA[2,4]-8	2336.58	2336.46
LsIA[2,4]-9	2553.85	2553.97

Table S3. Secondary structure contents of LsIA and its analogs. The secondary structure content was calculated by the Contin-LL program (Provencher & Glockner Method).[1]

peptide	Secondary Structures			
	α-Helix	β-Sheet	β-Turns	Random Coil
LsIA	19%	20%	27%	35%
LsIA [2,4]-1	17%	26%	24%	33%
LsIA [2,4]-2	10%	33%	24%	33%
LsIA [2,4]-3	13%	30%	24%	32%
LsIA [1,3]-1	16%	25%	25%	35%

2. Synthesis of Linker 4~6, AMC derivative and CPT derivatives

2.1. General procedures and apparatus

All chemical reagents for synthesis were purchased from commercial sources with a minimum purity of 95% and used without further purification. All reactions were monitored till completion using thin-layer chromatography (TLC) by Merck DC-plates (aluminum-based, silica gel 60 F_{254}, Merck KGaA). Visualization of TLC was performed by UV light (254 and 356 nm) and 5% phosphomolybdic acid in ethanol. Column chromatography with an appropriate solvent system as eluent was performed using 200-300 or 300-400 mesh silica gel. Mass spectra were obtained on

ESI-MS (Acquity H Class-Xevo TOD, Waters, Milford, MA, USA). Reversed-phase high-performance liquid chromatography (RP-HPLC) analysis was performed using a Waters e2695 system and a UV/visible detector (Waters 2489) with a Waters XBridge C18 column (250 × 4.6 mm, 5 µm, Waters, Milford, MA, USA). The chromatographic conditions were 50% to 100% buffer B in buffer A with a flow rate of 1 mL/min at UV-254 nm over 10 min, and the column temperature was 40 °C. A = 0.1% trifluoroacetic acid in H_2O and B = 0.1% trifluoroacetic acid in methanol. ^{1}H NMR spectra were recorded by 400 MHz frequency, and ^{13}C NMR was recorded at 100 MHz frequency on Bruker NMR spectrometer in the specified deuterated solvents.

2.2. Synthesis and characterization

Compounds **4** and **5** were synthesized according to the reported procedures.[2, 3]

Scheme S1. Synthetic route of Linker 6. *Reagents and conditions:* (a) 3-bromopropyne, K_2CO_3, acetone, 70 °C, 24 h; (b) $LiAlH_4$, THF, -10 °C, 1 h, 80 °C, 24 h; (c) PBr_3, DCM, rt, 24 h.

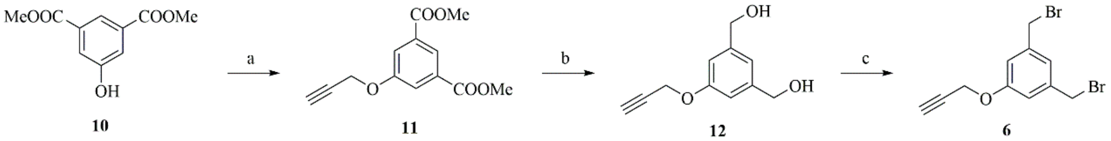

Dimethyl 5-(prop-2-yn-1-yloxy)isophthalate **11**. K_2CO_3 (1.32 g, 9.52 mmol) was added to a solution of Dimethyl 5- hydroxyisophthalate **10** (1.0 g, 4.76 mmol) and 3-bromopropyne (410 µL, 4.76 mmol) in acetone (20 mL). The reaction mixture was heated at 70 °C with stirring for 24 h under an argon(Ar) atmosphere and then cooled to room temperature. After removing undesirable precipitate by centrifugation (10000 g for 10 min at 25 °C), the solution was collected, and the solvent was removed by rotary evaporation. The product was washed with ethanol (5 × 5 mL), and dried under vacuum to afford **11** (1.06 g, 89.8%) as a white solid, which was used directly for the next step.

(5-(prop-2-yn-1-yloxy)-1,3-phenylene)dimethanol **12**. **11** (1 g, 4.03 mmol) was dissolved in tetrahydrofuran (20 mL) and cooled to -10 °C. $LiAlH_4$ (449 mg, 11.84 mmol) was added portionwise for 1 h with stirring under Ar. The mixture was then heated to 80 °C and reacted for another 24 h. After cooling to room temperature, the unreacted $LiAlH_4$ was quenched by $Na_2SO_4 \cdot 10H_2O$ until no gas emerged. After removing undesirable precipitate by centrifugation (10000 g for 10 min at 25°C), the solution was collected, and the solvent was removed by rotary evaporation to give **12** (550 mg, 71.0%) as a white solid which was used directly for the next step.

1,3-bis(bromomethyl)-5-(prop-2-yn-1-yloxy)benzene **6**.[4] **12** (1 g, 5.2 mmol) was dissolved in dichloromethane (20 mL), and the mixture was cooled to -10 °C. PBr_3 (1.96 mL, 20.8 mmol) was added dropwise with stirring in 10 min, and the reaction was stirred at room temperature for 24 h. After cooling to -10 °C, residual PBr_3 was quenched by saturated $NaHCO_3$ solution. The resulting organic layer was washed with water (3 × 5 mL), dried by anhydrous Na_2SO_4, and evaporated to give **6** (1.19 g, 71.9%) as a white solid. ^{1}H NMR (400 MHz, Chloroform-d) δ 7.05 (s, 1H), 6.94 (s, 2H), 4.71 (d, J = 2.4 Hz, 2H), 4.44 (s, 4H), 2.55 (t, J = 2.4 Hz, 1H).

Scheme S2. Synthetic routes of derivatized fluorescence and drug. *Reagents and conditions:* (a) succinic anhydride, DMF, 60 °C, 24 h; (b) Azido-PEG3-NH$_2$, EDCI, HOBT, DIPEA, DMF, rt, 16 h; (c) CuSO$_4$·5H$_2$O, sodium ascorbate, H$_2$O, MeCN, rt, overnight; (d) azidoacetic acid, EDCI, DMAP, DCM, rt, overnight; (e) CuSO$_4$·5H$_2$O, sodium ascorbate, H$_2$O, MeCN, rt, overnight; (f) succinic anhydride, 0 °C, 15 min, DBU, DCM, 4 h; (g) Azido-PEG3-NH$_2$, HATU, DIPEA, DCM, rt, 10 h; (h) CuSO$_4$·5H$_2$O, sodium ascorbate, H$_2$O, MeCN, rt, overnight.

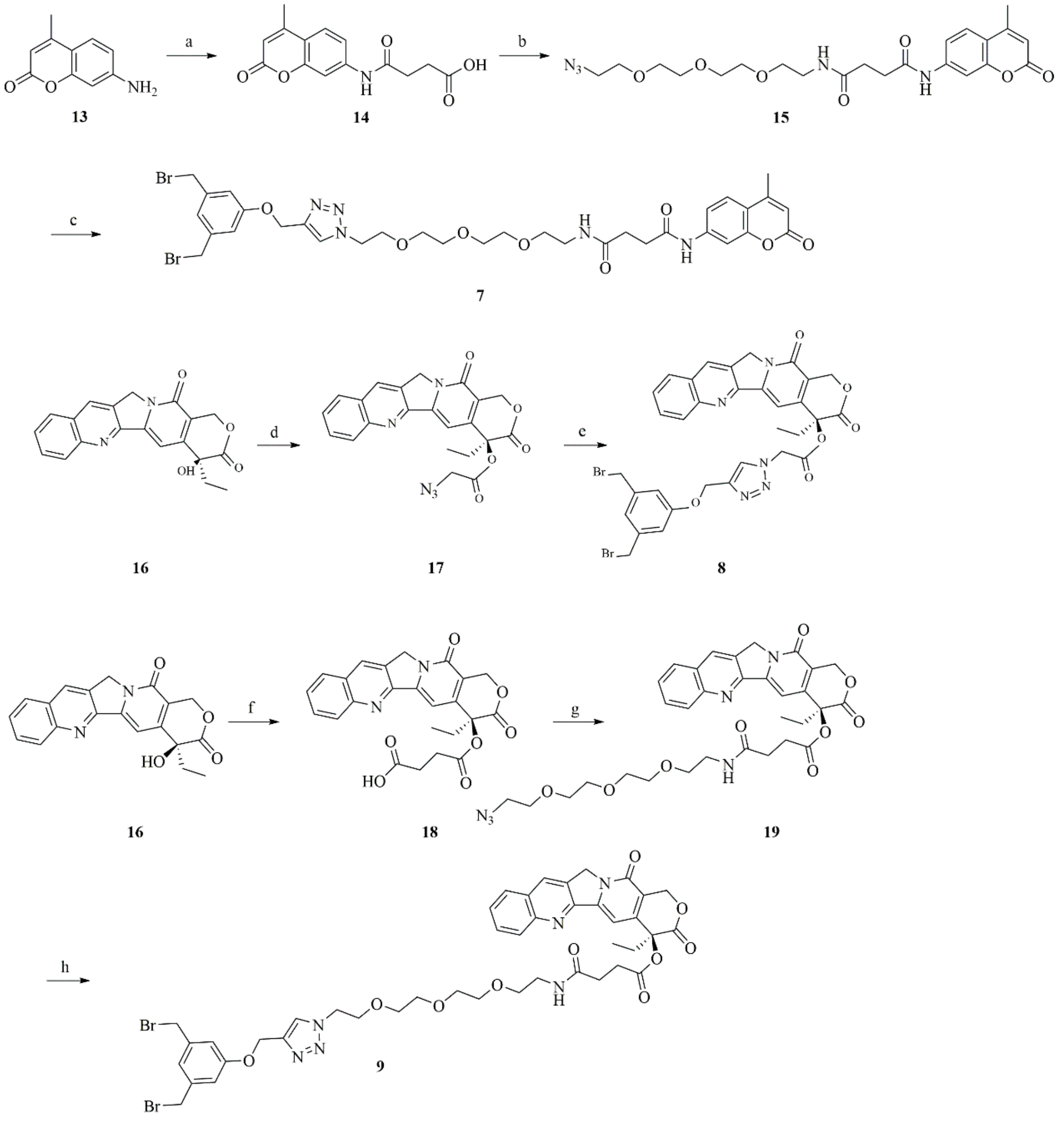

4-((4-methyl-2-oxo-2H-chromen-7-yl)amino)-4-oxobutanoic acid **14**.[5] Coumarin 120 (AMC) **13** (100 mg, 571 μmol) and succinic anhydride (57.1 mg, 571 μmol) were dissolved in DMF (1 mL), and the mixture was stirred at 60 °C for 24 h. After cooling to room temperature, the reaction mixture was dried under vacuum. The residue was sonicated in EtOH (100%, 2 mL) for 1 min and then filtered to give **14** (123.6 mg, 78.7%) as a brown solid; ^{1}H NMR (400 MHz, DMSO-d6) δ 12.23 (s, 1H), 10.57 (s, 1H), 7.76 (s, 1H), 7.69 (d, J = 8.7 Hz, 1H), 7.49 (d, J = 8.7 Hz, 1H), 6.24 (s, 1H), 2.62 (t, J = 6.6 Hz, 2H), 2.55 (d, J = 6.4 Hz, 2H), 2.39 (s, 3H); ^{13}C NMR (100 MHz, DMSO) δ 173.74, 170.98, 160.06, 153.69, 153.13, 142.61, 125.88, 114.96, 114.74, 112.08, 105.32, 31.24, 28.61, 17.97.

N[1]-(2-(2-(2-(2-azidoethoxy)ethoxy)ethoxy)ethyl)-N[4]-(4-methyl-2-oxo-2H-chromen-7-yl)-succinamide **15**. EDCI (209 mg, 1.09 mmol), HOBT (147 mg, 1.09 mmol) and DIPEA(380 μL, 2.18 mmol) were added to a solution of **14** (100 mg, 363 μmol) and Azido-PEG3-NH_2 (72.1 μL, 363 μmol) in DMF (2 mL). The reaction mixture was stirred at room temperature for 16 h and then evaporated in vacuo. The solid obtained was purified by column chromatography (CH_2Cl_2/MeOH (v/v) = 50/1) to afford pure **15** (35.2 mg, 20.4%) as a brown solid; ^{1}H NMR (400 MHz, Chloroform-d) δ 9.69 (s, 1H), 7.68 (d, J = 2.0 Hz, 1H), 7.39 (d, J = 8.7 Hz, 1H), 7.33 – 7.28 (m, 1H), 6.77 (t, J = 5.4 Hz, 1H), 6.09 (d, J = 1.6 Hz, 1H), 3.68 – 3.62 (m, 10H), 3.57 (t, J = 5.0 Hz, 2H), 3.47 (q, J = 5.1 Hz, 2H), 3.38 (t, J = 5.0 Hz, 2H), 2.76 (dd, J = 8.1, 4.7 Hz, 2H), 2.67 (dd, J = 8.0, 4.7 Hz, 2H), 2.35 (s, 3H); ^{13}C NMR (100 MHz, $CDCl_3$) δ 172.87, 171.37, 161.36, 154.15, 152.62, 142.16, 124.94, 115.63, 115.54, 112.98, 106.95, 70.74, 70.72, 70.61, 70.38, 70.06, 69.66, 50.79, 39.71, 32.66, 31.00, 18.57; Rt = 4.285 min; exact mass (electrospray) m/z calcd for $C_{22}H_{29}N_5O_7$ $(M + H)^+$ 476.2097, found 476.2149.

N[1]-(2-(2-(2-(2-(4-((3,5-bis(bromomethyl)phenoxy)methyl)-1H-1,2,3-triazol-1-yl)ethoxy)-ethoxy)ethoxy)ethyl)-N[4]-(4-methyl-2-oxo-2H-chromen-7-yl)succinamide **7**. A solution of $CuSO_4 \cdot 5H_2O$ (7.88 mg, 31.5 μmol) and sodium ascorbate (12.5 mg, 63.1 μmol) in water (200 μL) was added to a stirred solution of **15** (10 mg, 21.0 μmol) and **6** (8.03 mg, 25.2 μmol) in MeCN (1 mL). The reaction mixture was stirred at room temperature overnight and then evaporated in vacuo. The solid obtained was purified by column chromatography (CH_2Cl_2/MeOH (v/v) = 25/1) to afford **7** (8.5 mg, 50.9%) as a brown solid, which was used directly in the next step; Rt = 6.418 min; exact mass (electrospray) m/z calcd for $C_{33}H_{39}Br_2N_5O_8$$(M + H)^+$ 794.1177, found 794.1261.

(S)-4-ethyl-3,14-dioxo-3,4,12,14-tetrahydro-1H-pyrano[3',4':6,7]indolizino[1,2-b]quinolin-4-yl 2-azidoacetate **17**. EDCI (165 mg, 861 μmol) and DMAP (105 mg, 861 μmol) were added to a solution of **16** (100 mg, 287 μmol) and azidoacetic acid (21.5 μL, 287 μmol) in DCM (1 mL). The reaction mixture was stirred at room temperature overnight and then evaporated in vacuo. The solid obtained was purified by column chromatography (CH_2Cl_2/MeOH (v/v) = 100/1) to afford **17** (55.3 mg, 44.7%) as a yellow solid. ^{1}H NMR (400 MHz, Chloroform-d : Methanol-d4=2:1) δ 8.54 (s, 1H), 8.19 (d, J = 8.5 Hz, 1H), 8.05 – 7.92 (m, 1H), 7.86 (ddd, J = 8.5, 6.9, 1.5 Hz, 1H), 7.69 (ddd, J = 8.1, 6.9, 1.2 Hz, 1H), 7.40 (s, 1H), 5.53 (dd, J = 107.4, 17.1 Hz, 2H), 5.30 (d, J = 2.1 Hz, 2H), 4.18 (d, J = 1.5 Hz, 2H), 2.30 – 2.10 (m, 2H), 1.01 (t, J = 7.5 Hz, 3H); ^{13}C NMR (100 MHz, $CDCl_3$: CD_3OD=2:1) δ 168.29, 167.82, 157.89, 151.85, 148.21, 146.36, 146.34, 133.08, 131.80, 129.24, 129.23, 128.91, 128.90, 128.87, 120.32, 97.44, 77.66, 67.25, 50.64, 50.24, 31.79, 7.75; Rt = 6.311 min; exact mass (electrospray) m/z calcd for $C_{22}H_{17}N_5O_5$ $(M + H)^+$ 432.1259, found 432.1301.

(S)-4-ethyl-3,14-dioxo-3,4,12,14-tetrahydro-1H-pyrano[3',4':6,7]indolizino[1,2-b]quinolin-4-yl 2-(4-((3,5-bis(bromomethyl)phenoxy)methyl)-1H-1,2,3-triazol-1-yl)acetate **8**. A solution of $CuSO_4 \cdot 5H_2O$ (8.68 mg, 34.8 μmol) and sodium ascorbate (13.8 mg, 69.5 μmol) in water (200 μL) was added to a stirred solution of **17** (10 mg, 23.2 μmol) and **6** (8.85 mg, 27.8 μmol) in MeCN (1 mL). The reaction mixture was stirred at room temperature overnight and then evaporated in vacuo. The solid obtained was purified by column chromatography (CH_2Cl_2/MeOH (v/v) = 100/1) to afford **8** (10.6 mg, 60.4 %) as a yellow solid, which was used directly in the next step; Rt = 8.382 min; exact mass (electrospray) m/z calcd for $C_{33}H_{27}Br_2N_5O_6$ $(M + H)^+$ 750.0340, found 750.0361.

(S)-4-((4-ethyl-3,14-dioxo-3,4,12,14-tetrahydro-1H-pyrano[3',4':6,7]indolizino[1,2-b]quinolin-4-yl)oxy)-4-oxobutanoic acid **18**. [6] A solution of **16** (100 mg, 287 μmol) and succinic anhydride (86.2 mg, 861 μmol) in DCM (4.3 mL) was stirred under an ice bath for 15 min under Ar atmosphere. Then DBU (129 μL, 861 μmol) in DCM (1.3 mL) was added dropwise. The mixture was reacted for another 4 hours. The reaction was dried under vacuum to get the orange solid (186 mg), which was used directly for the next step.

(S)-4-ethyl-3,14-dioxo-3,4,12,14-tetrahydro-1H-pyrano[3',4':6,7]indolizino[1,2-b]quinolin-4-yl 1-azido-13-oxo-3,6,9-trioxa-12-azahexadecan-16-oate **19**. To a solution of unpurified **18** (185 mg, 412 μmol) and Azido-PEG3-NH_2 (45.5 μL, 229 μmol) in DCM (10 mL) were added HATU (261 mg, 687 μmol) and DIPEA (200 μL, 1.15 mmol). The reaction mixture was stirred at room temperature for 10 h and then evaporated in vacuo. The solid obtained was purified by column chromatography (CH_2Cl_2/MeOH (v/v) = 50/1) to afford **19** (97.9 mg, 65.9%) as an orange solid; ^1H NMR (400 MHz, Chloroform-d) δ 8.44 (s, 1H), 8.32 (d, J = 8.5 Hz, 1H), 7.95 (d, J = 8.2 Hz, 1H), 7.88 – 7.81 (m, 1H), 7.68 (t, J = 7.5 Hz, 1H), 7.45 (s, 1H), 6.38 (s, 1H), 5.65 (d, J = 17.2 Hz, 1H), 5.36 (d, J = 17.2 Hz, 1H), 5.26 (s, 2H), 3.62 (d, J = 2.6 Hz, 6H), 3.59 (dd, J = 5.9, 3.2 Hz, 2H), 3.56 – 3.52 (m, 2H), 3.46 (dd, J = 7.8, 3.5 Hz, 2H), 3.40 (td, J = 10.8, 9.8, 4.8 Hz, 2H), 3.34 (t, J = 5.1 Hz, 2H), 2.99 – 2.76 (m, 2H), 2.59 – 2.44 (m, 2H), 2.18 (ddq, J = 46.9, 14.4, 7.4 Hz, 2H), 0.98 (t, J = 7.4 Hz, 3H); ^{13}C NMR (100 MHz, $CDCl_3$) δ 172.06, 170.96, 167.51, 157.34, 151.73, 147.73, 146.28, 145.38, 132.33, 131.35, 128.85, 128.78, 128.44, 128.35, 128.28, 120.49, 97.53, 76.17, 70.74, 70.66, 70.62, 70.29, 70.08, 69.78, 67.02, 50.75, 50.07, 39.43, 31.77, 30.85, 29.62, 7.73; Rt = 5.013 min; exact mass (electrospray) m/z calcd for $C_{32}H_{36}N_6O_9$ $(M + H)^+$ 649.2574, found 649.2577.

(S)-4-ethyl-3,14-dioxo-3,4,12,14-tetrahydro-1H-pyrano[3',4':6,7]indolizino[1,2-b]quinolin-4-yl 1-(4-((3,5-bis(bromomethyl)phenoxy)methyl)-1H-1,2,3-triazol-1-yl)-13-oxo-3,6,9-trioxa-12-azahexadecan-16-oate **9**. A solution of $CuSO_4 \cdot 5H_2O$ (5.77 mg, 23.1 μmol) and sodium ascorbate (9.16 mg, 46.2 μmol) in water (200 μL) was added to a stirred solution of **19** (10 mg, 15.4 μmol) and **6** (5.88 mg, 18.5 μmol) in MeCN (1 mL). The reaction mixture was stirred at room temperature overnight and then evaporated in vacuo. The solid obtained was purified by column chromatography (CH_2Cl_2/MeOH (v/v) = 50/1) to afford **9** (8.6 mg, 57.7%) as an orange solid, which was used directly in the next step; Rt = 7.119 min; exact mass (electrospray) m/z calcd for $C_{43}H_{46}Br_2N_6O_{10}$ $(M + H)^+$ 967.1655, found 967.1699.

2.3. HPLC, Mass and NMR analyses

Spectral data of compound **5**

^{1}H-NMR (Acetone-d, 400 MHz, δ ppm) spectrum of compound **5**

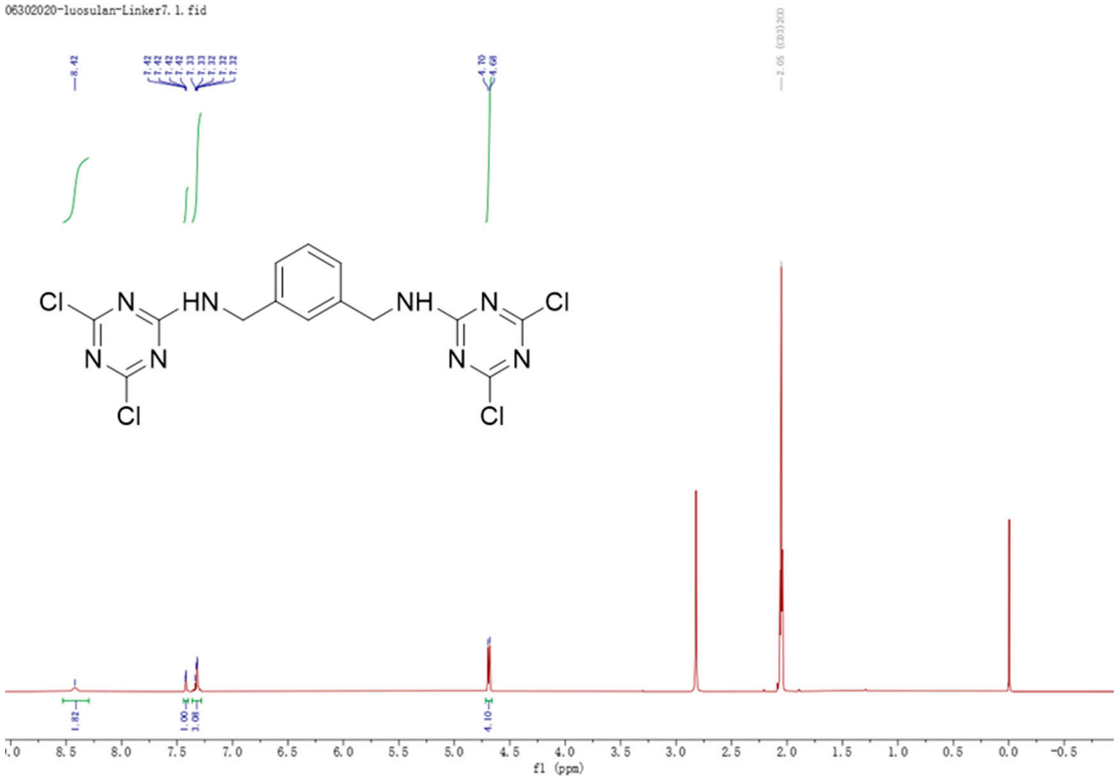

Spectral data1 of compound **6**

^{1}H-NMR (Chloroform-d, 400 MHz, δ ppm) spectrum of compound **6**

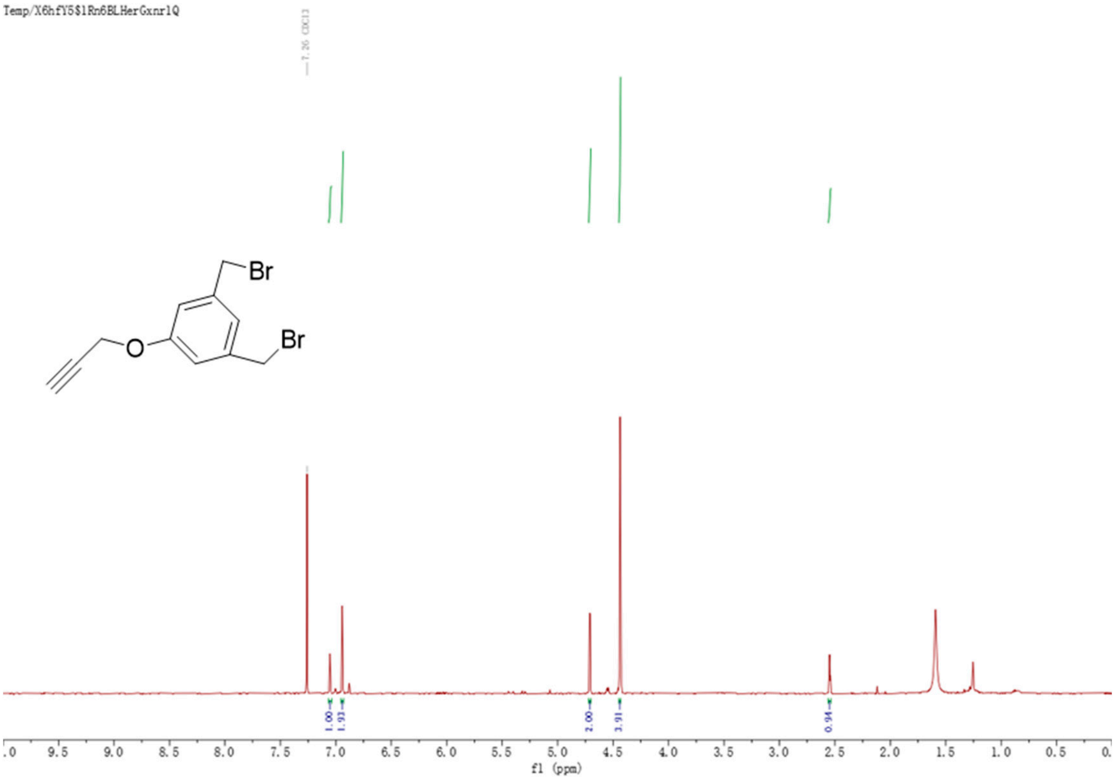

Spectral data of compound **7**

HPLC and mass spectrum of compound **7**

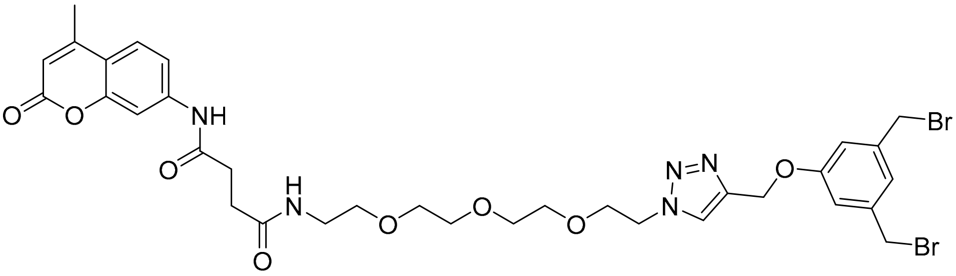

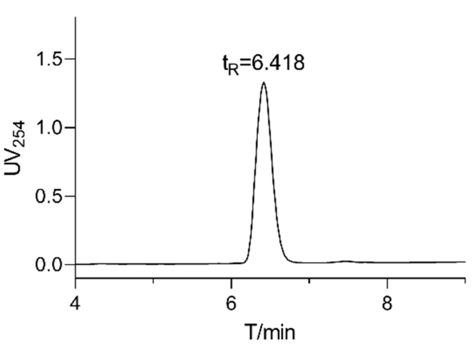

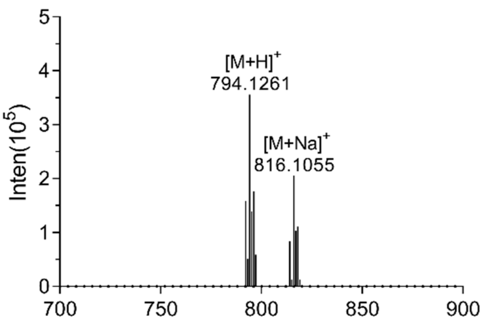

Spectral data of compound **8**

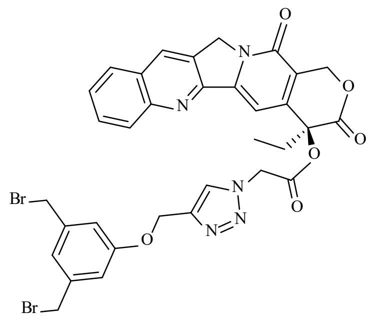

HPLC and mass spectrum of compound **8**

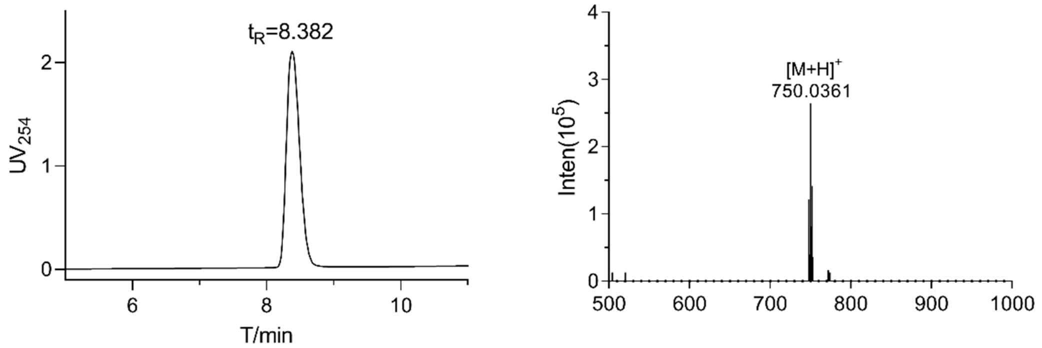

Spectral data of compound **9**

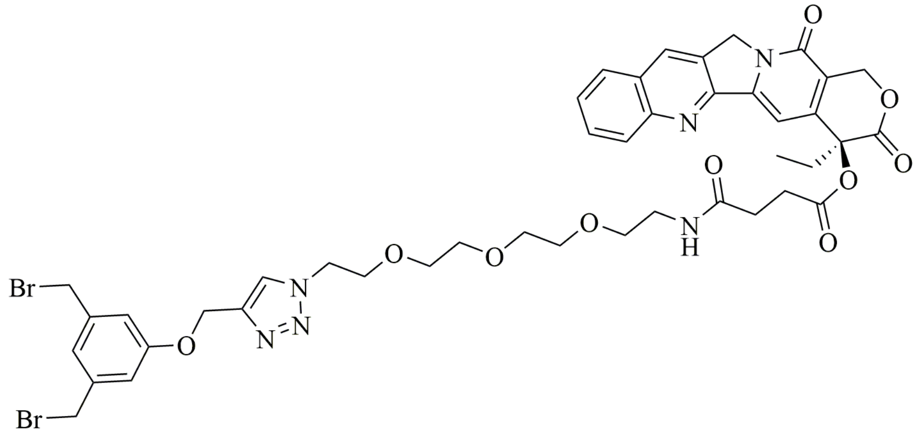

HPLC and mass spectrum of compound **9**

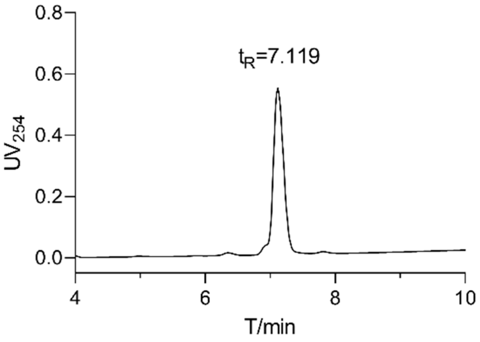

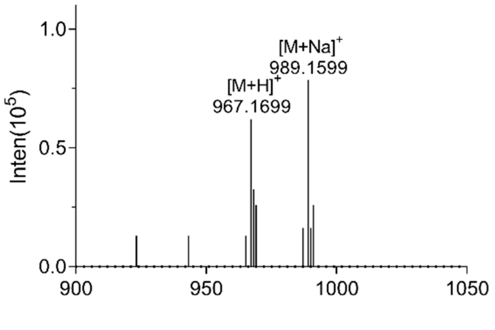

Spectral data of compound **14**

^{1}H-NMR (DMSO-d6, 400 MHz, δ ppm) spectrum of compound **14**

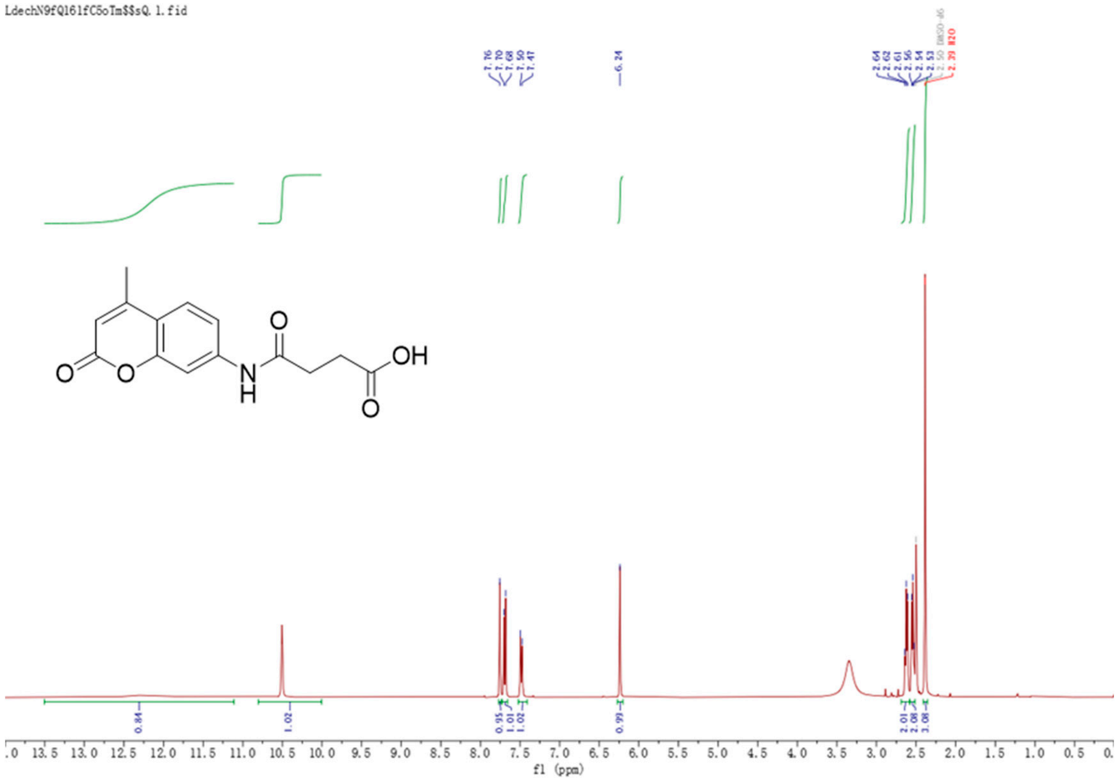

^{13}C-NMR (DMSO-d6, 100 MHz, δ ppm) spectrum of compound **14**

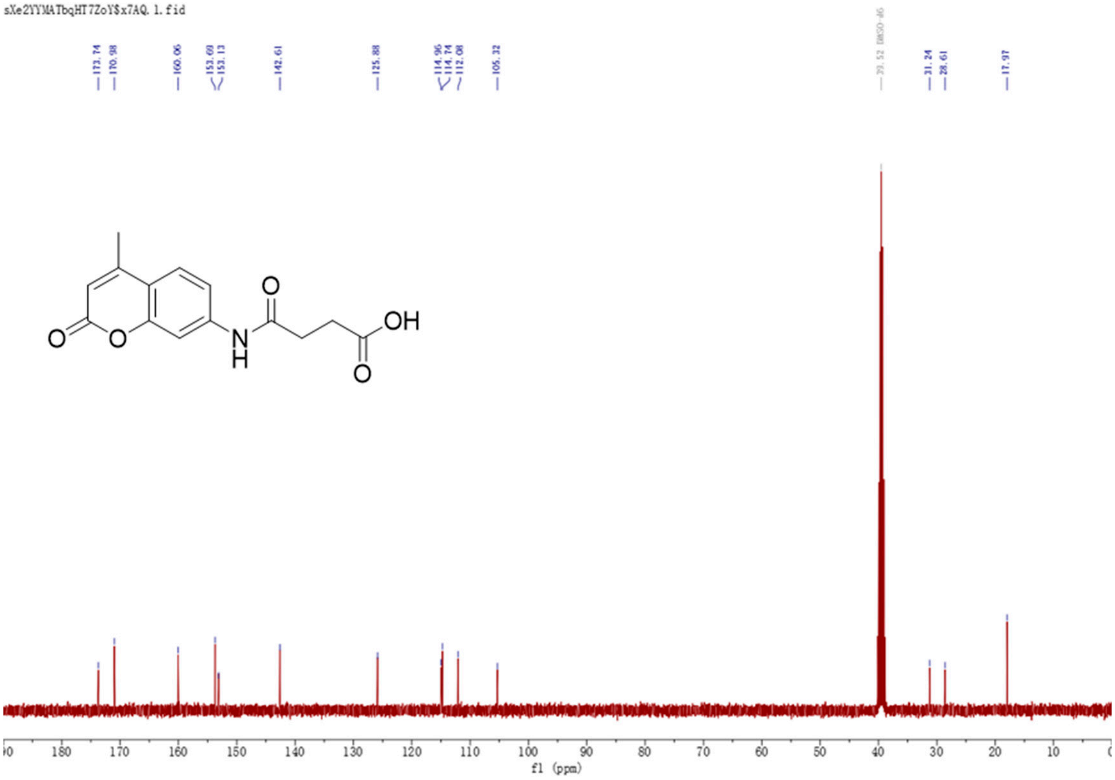

Spectral data of compound **15**

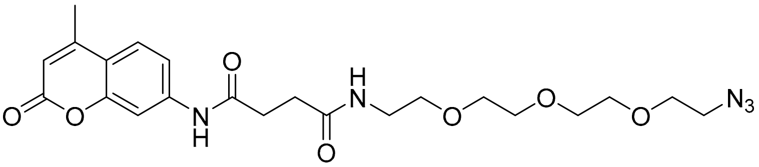

HPLC and mass spectrum of compound **15**

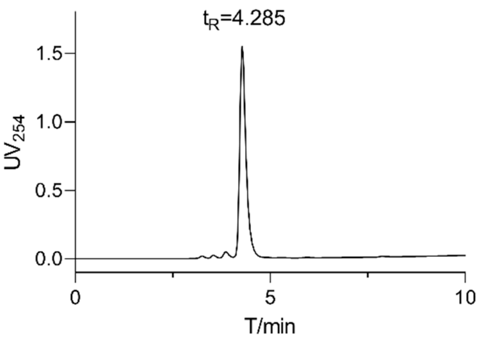

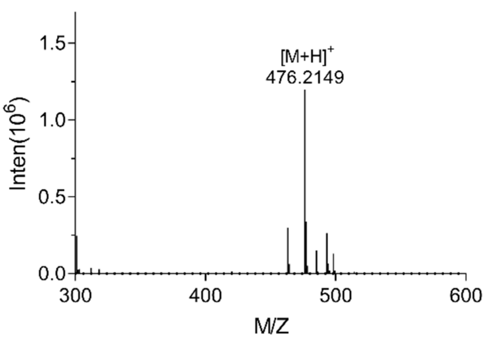

^{1}H-NMR (Chloroform-d, 400 MHz, δ ppm) spectrum of compound **15**

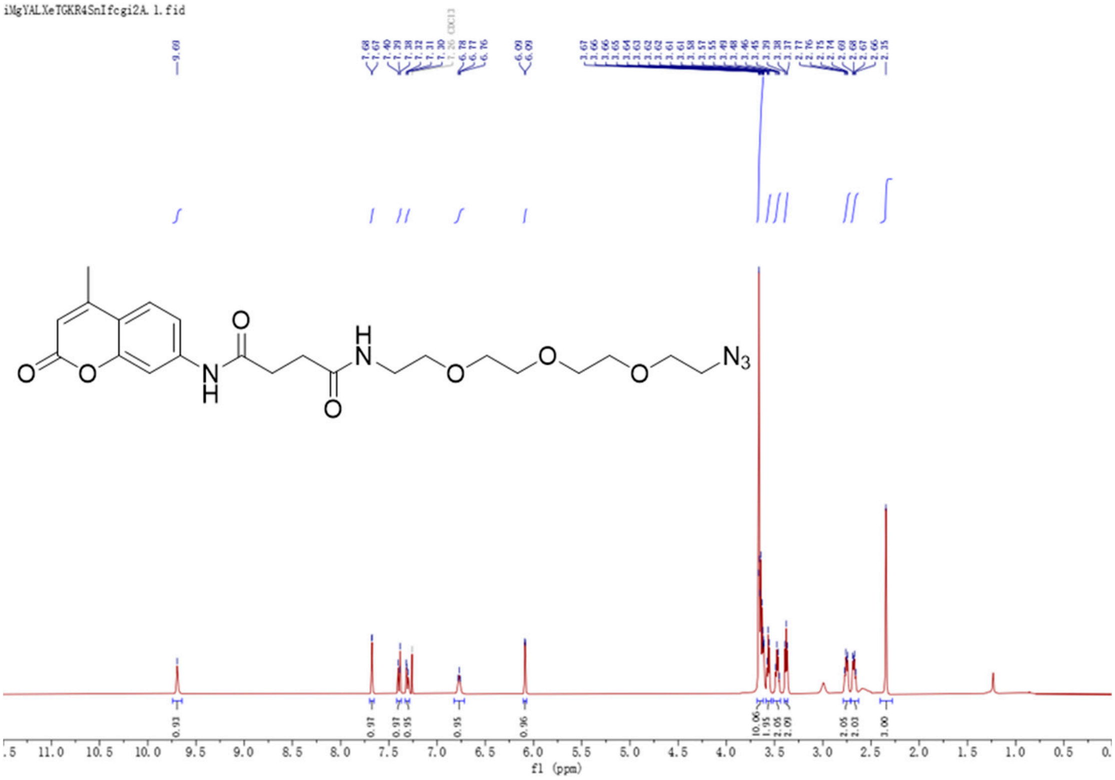

^{13}C-NMR (Chloroform-d, 100 MHz, δ ppm) spectrum of compound **15**

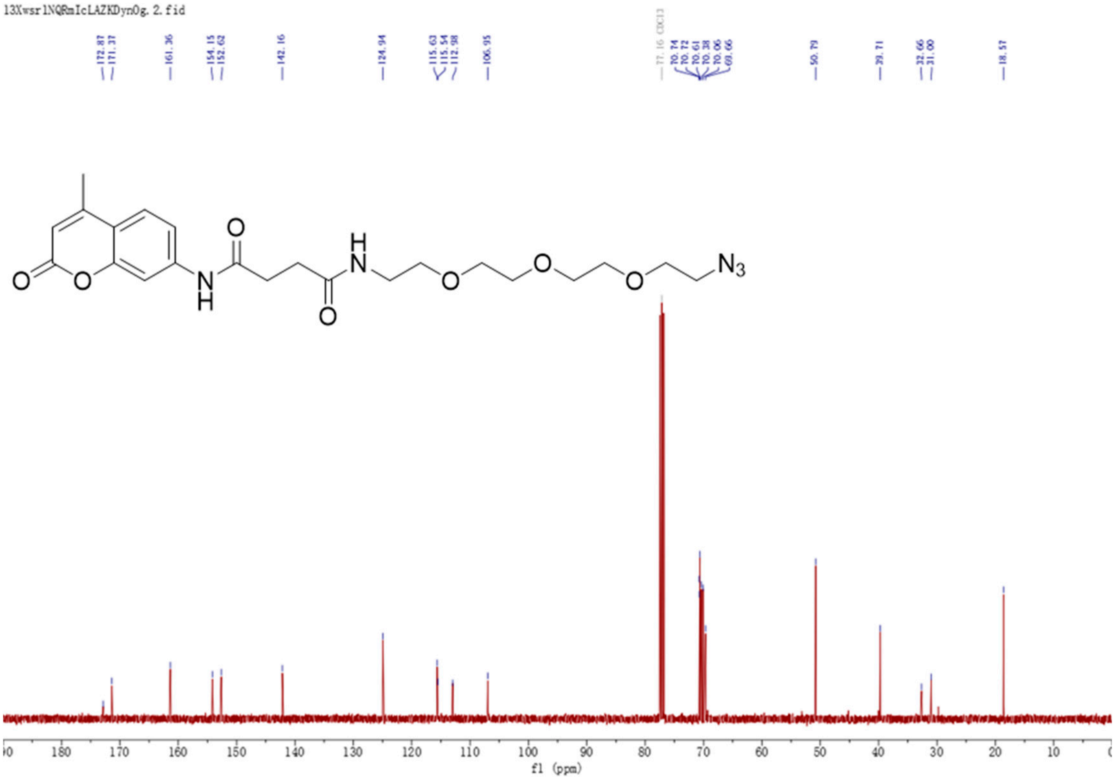

Spectral data of compound **17**

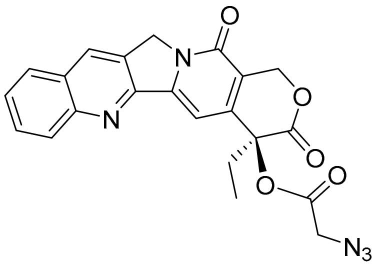

HPLC and mass spectrum of compound **17**

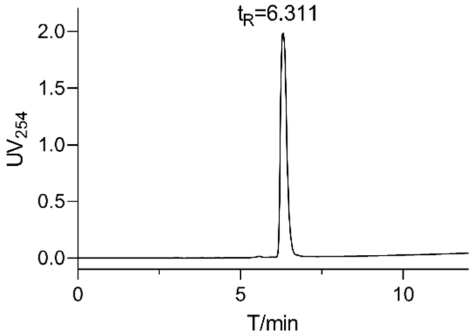

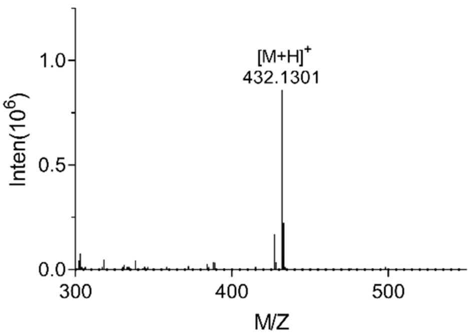

^{1}H-NMR (Chloroform-d : Methanol-d4=2:1, 400 MHz, δ ppm) spectrum of compound **17**

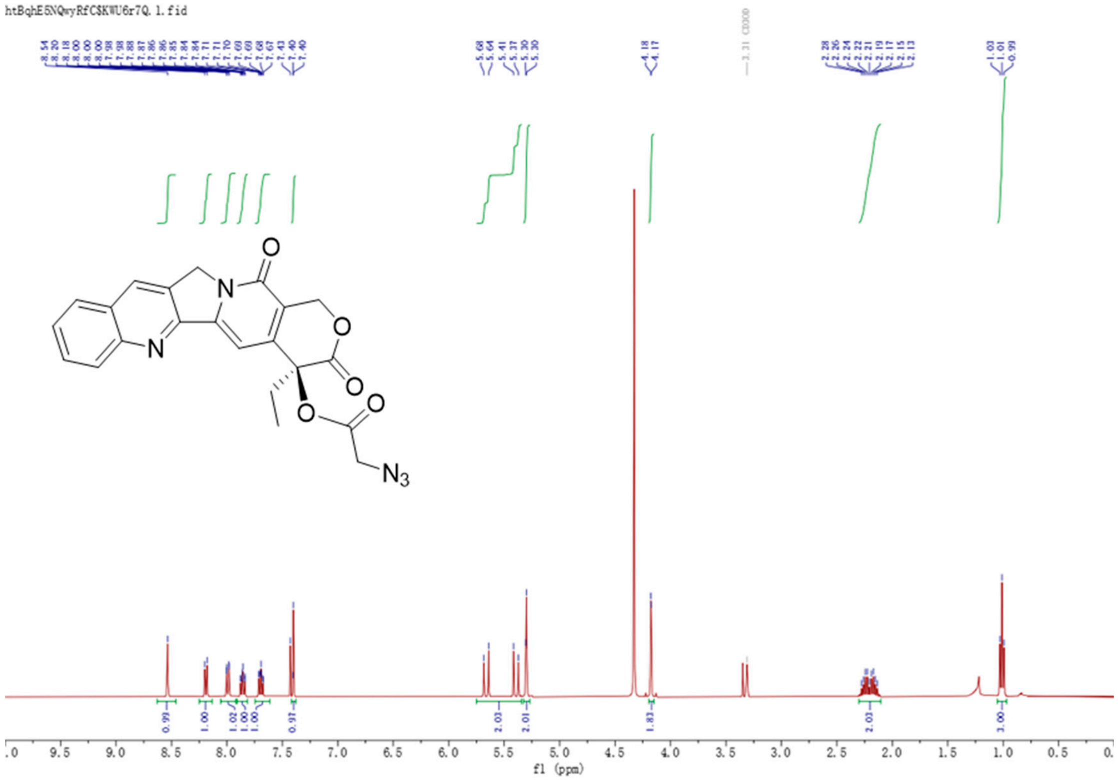

^{13}C-NMR (Chloroform-d : Methanol-d4=2:1, 100 MHz, δ ppm) spectrum of compound **17**

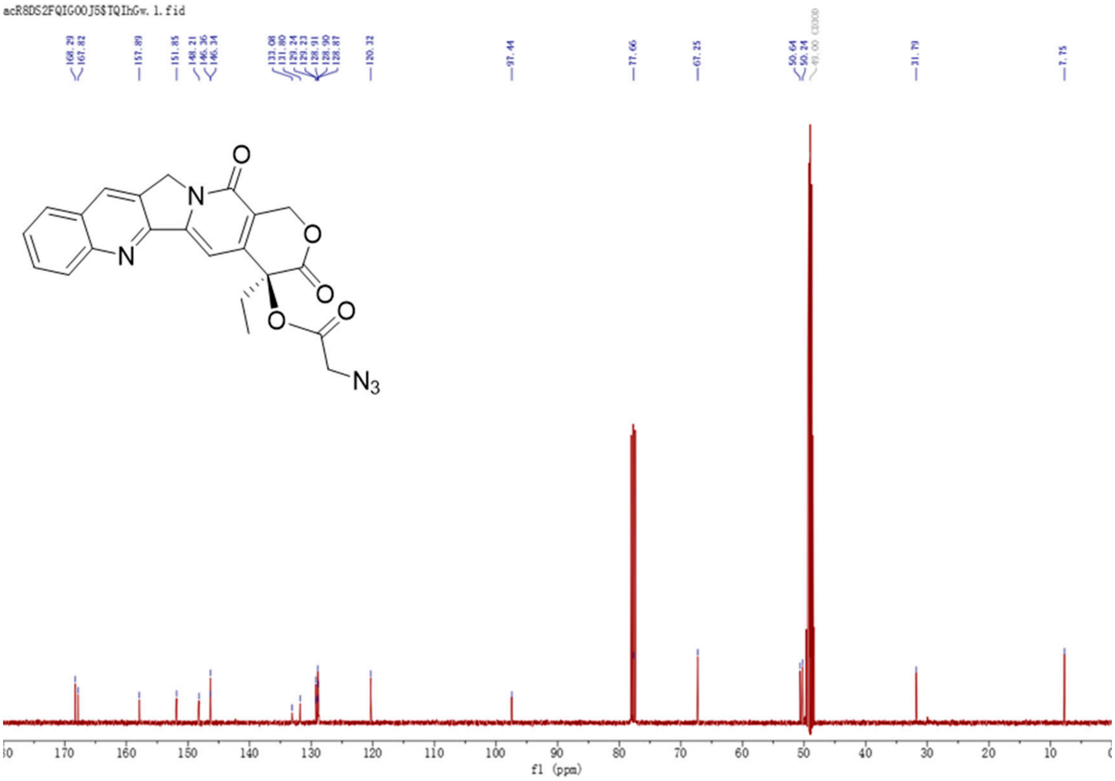

Spectral data of compound **19**

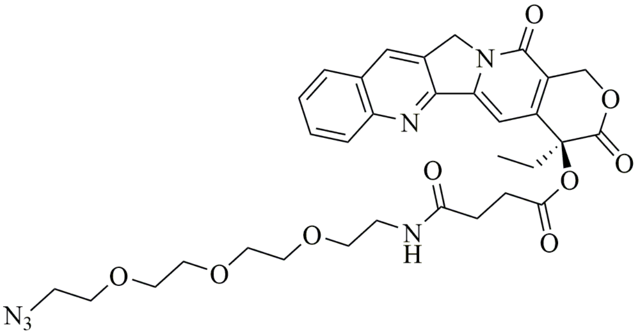

HPLC and mass spectrum of compound **19**

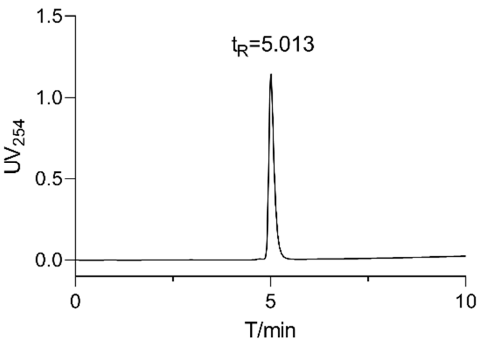

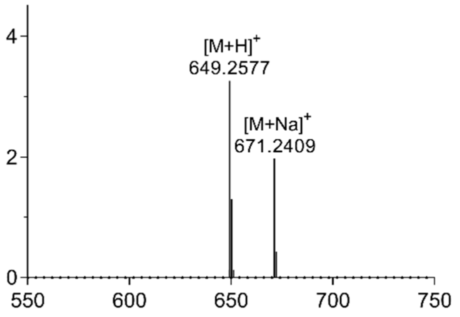

^{1}H-NMR (Chloroform-d, 400 MHz, δ ppm) spectrum of compound **19**

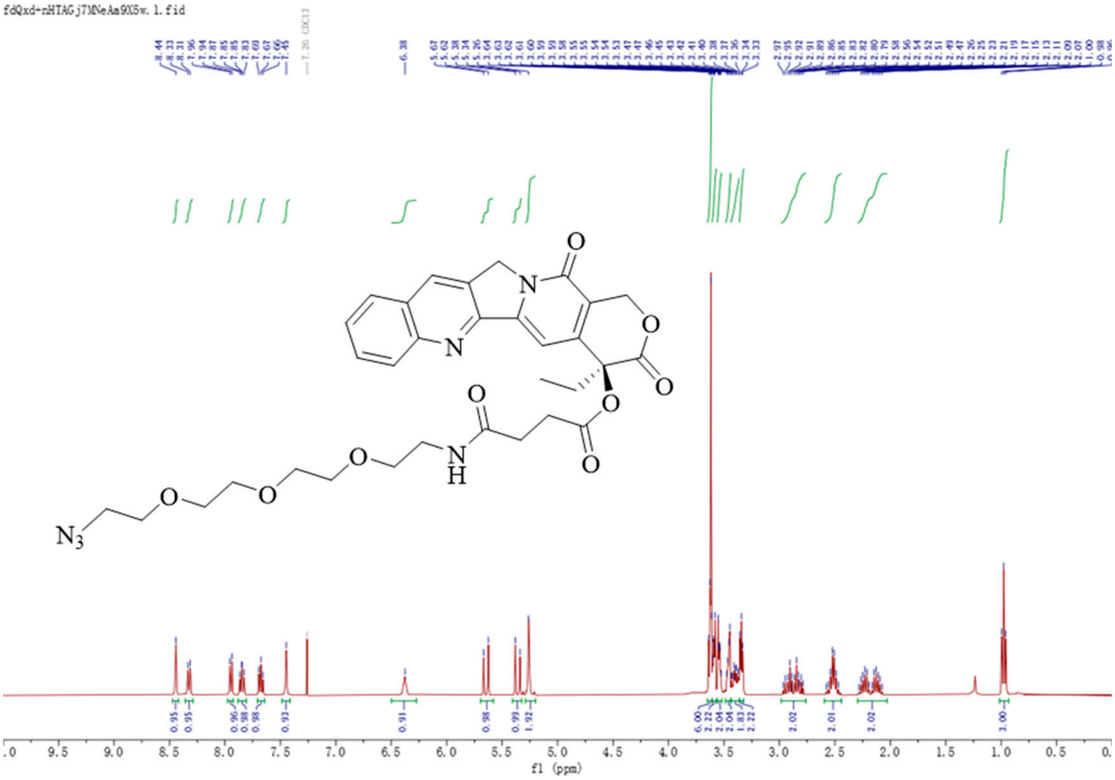

^{13}C-NMR (Chloroform-d, 100 MHz, δ ppm) spectrum of compound **19**

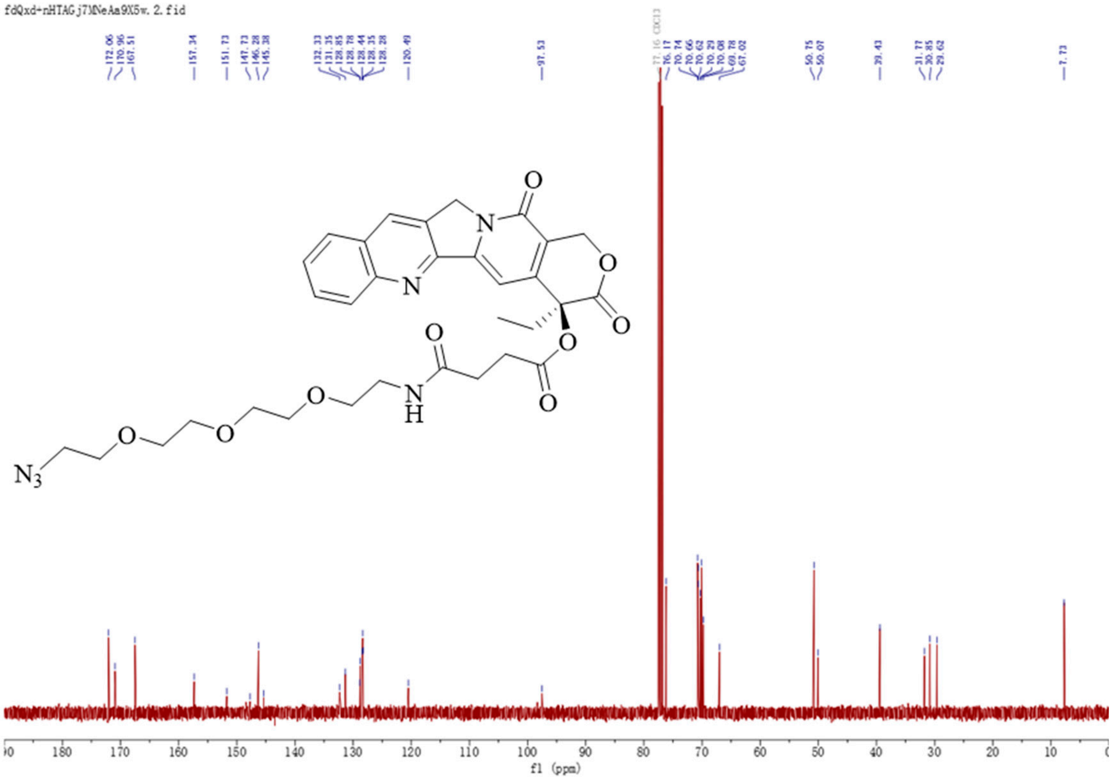

3. References

1. Whitmore, L.; Wallace, B. A. Protein secondary structure analyses from circular dichroism spectroscopy: methods and reference databases. *Biopolymers* **2008,** *89*, 392-400.

2. Li, Z.; Huang, R.; Xu, H.; Chen, J.; Zhan, Y.; Zhou, X.; Chen, H.; Jiang, B. Divinylsulfonamides as Specific Linkers for Stapling Disulfide Bonds in Peptides. *Org Lett* **2017,** *19*, 4972-4975.

3. Lautrette, G.; Touti, F.; Lee, H. G.; Dai, P.; Pentelute, B. L. Nitrogen Arylation for Macrocyclization of Unprotected Peptides. *J Am Chem Soc* **2016,** *138*, 8340-3.

4. Wang, Y.; Zhang, D.; Liang, X.; Shehzad, M. A.; Xiao, X.; Zhu, Y.; Ge, X.; Zhang, J.; Ge, Z.; Wu, L.; Xu, T. Improving fuel cell performance of an anion exchange membrane by terminal pending bis-cations on a flexible side chain. *J. Membr. Sci.* **2020,** *595*, 117483.

5. Bossmann, S.; Leaym, X.; Kraft, S. Synthesis of Water-Soluble Highly Charged and Methylene-Bridged -Resorcin[4]arenes. *Synthesis* **2008,** *6*, 932-942.

6. Wang, J. L.; Wang, K. X.; Han, T. L.; Li, J. M.; He, X.; Rong, R. X.; Cao, Z. R.; Li, X. L.; Wang, K. R. Antitumour properties based on the self-assembly of camptothecin and carbamoylmannose conjugates. *Chem Biol Drug Des* **2020,** *96*, 870-877.
